# TCR-mimic bispecific antibodies to target the HIV-1 reservoir

**DOI:** 10.1073/pnas.2123406119

**Published:** 2022-04-08

**Authors:** Srona Sengupta, Nathan L. Board, Fengting Wu, Milica Moskovljevic, Jacqueline Douglass, Josephine Zhang, Bruce R. Reinhold, Jonathan Duke-Cohan, Jeanna Yu, Madison C. Reed, Yasmine Tabdili, Aitana Azurmendi, Emily J. Fray, Hao Zhang, Emily Han-Chung Hsiue, Katharine Jenike, Ya-Chi Ho, Sandra B. Gabelli, Kenneth W. Kinzler, Bert Vogelstein, Shibin Zhou, Janet D. Siliciano, Scheherazade Sadegh-Nasseri, Ellis L. Reinherz, Robert F. Siliciano

**Affiliations:** ^a^Department of Medicine, The Johns Hopkins University School of Medicine, Baltimore, MD 21205;; ^b^Department of Pathology, The Johns Hopkins University School of Medicine, Baltimore, MD 21205;; ^c^Ludwig Center, Sidney Kimmel Comprehensive Cancer Center, The Johns Hopkins University School of Medicine, Baltimore, MD 21287;; ^d^Lustgarten Pancreatic Cancer Research Laboratory, Sidney Kimmel Comprehensive Cancer Center, The Johns Hopkins University School of Medicine, Baltimore, MD 21287;; ^e^Laboratory of Immunobiology, Dana-Farber Cancer Institute, Boston, MA 02115;; ^f^Department of Medical Oncology, Dana-Farber Cancer Institute, Boston, MA 02115;; ^g^Department of Medicine, Harvard Medical School, Boston, MA 02115;; ^h^Department of Biophysics and Biophysical Chemistry, The Johns Hopkins University School of Medicine, Baltimore, MD 21287;; ^i^Department of Molecular Microbiology and Immunology, The Johns Hopkins Bloomberg School of Public Health, Baltimore, MD 21205;; ^j^Department of Microbial Pathogenesis, Yale University School of Medicine, New Haven, CT 06519;; ^k^Bloomberg-Kimmel Institute for Cancer Immunotherapy, Sidney Kimmel Comprehensive Cancer Center, Baltimore, MD 21287;; ^l^HHMI, The Johns Hopkins University School of Medicine, Baltimore, MD 21205

**Keywords:** HIV-1, latent reservoir, immunotherapy, human leukocyte antigen, antigen processing

## Abstract

HIV-1 infection is incurable due to the persistence of the virus in a latent reservoir of resting memory CD4^+^ T cells. “Shock-and-kill” approaches that seek to induce HIV-1 gene expression, protein production, and subsequent targeting by the host immune system have been unsuccessful due to a lack of effective latency-reversing agents (LRAs) and kill strategies. In an effort to develop reagents that could be used to promote killing of infected cells, we constructed T cell receptor (TCR)-mimic antibodies to HIV-1 peptide-major histocompatibility complexes (pMHC). Using phage display, we panned for phages expressing antibody-like variable sequences that bound HIV-1 pMHC generated using the common HLA-A*02:01 allele. We targeted three epitopes in Gag and reverse transcriptase identified and quantified via Poisson detection mass spectrometry from cells infected in vitro with a pseudotyped HIV-1 reporter virus (NL4.3 dEnv). Sequences isolated from phages that bound these pMHC were cloned into a single-chain diabody backbone (scDb) sequence, such that one fragment is specific for an HIV-1 pMHC and the other fragment binds to CD3ε, an essential signal transduction subunit of the TCR. Thus, these antibodies utilize the sensitivity of T cell signaling as readouts for antigen processing and as agents to promote killing of infected cells. Notably, these scDbs are exquisitely sensitive and specific for the peptide portion of the pMHC. Most importantly, one scDb caused killing of infected cells presenting a naturally processed target pMHC. This work lays the foundation for a novel therapeutic killing strategy toward elimination of the HIV-1 reservoir.

## Significance

Novel approaches to promote killing of HIV-1–infected cells are necessary for elimination of the latent reservoir, the main barrier to a cure. Here, we utilized a diverse phage-display library to construct T cell receptor (TCR)-mimic antibodies to HIV-1 peptide-major histocompatibility complexes (pMHC). We show that single-chain diabody forms of these antibodies recognize distinct epitopes in Gag and reverse transcriptase in a specific manner and induce T cell-mediated killing of HIV-1–infected CD4^+^ T cells. This study lays the groundwork for future exploration of pMHC-based immunotherapeutic approaches toward elimination of the latent reservoir once effective latency-reversing strategies are developed.

HIV-1 is incurable due to a long-lived reservoir in resting CD4^+^ T cells that harbor latent, replication-competent proviruses (1–5). When resting CD4^+^ T cells are activated by antigen or other stimuli, the transcriptional environment becomes permissive for viral gene expression, and infectious virions can be released. If combination antiretroviral therapy (cART) is interrupted, exponential viral replication ensues (6, 7), eventually leading to acquired immune deficiency (AIDS). Due to the extremely slow decay of the latent reservoir (*t*_1/2_ ∼3.6 y), persons living with HIV-1 (PLWH) must remain on cART for life (8–11).

Efforts to cure HIV-1 infection have focused on the “shock-and-kill” strategy, which relies upon latency-reversing agents (LRAs) to induce viral gene expression, revealing these infected cells to the immune system (“shock”). These cells can then be targeted for cytolysis by CD8^+^ cytolytic T lymphocytes (CTL) or natural killer (NK) cells (“kill”) (12). CTL-mediated killing requires T cell receptor (TCR) recognition of short (8 to 11 amino acid) peptide fragments of HIV-1 proteins presented on major histocompatibility class I (MHC-I) molecules (13). In principle, shock and kill should reduce the reservoir. Yet, while certain LRAs have caused viral “blips” (transient increases in plasma virus into the detectable range) in PLWH on cART, current LRAs alone have not reduced the latent reservoir (14–17). One potential explanation is that CTL function is compromised in PLWH and is not fully restored by cART (18, 19). Recent studies have shown that prestimulation of CTLs is required for elimination of infected cells following latency reversal (20). Additionally, the latent reservoir of PLWH contains proviruses with escape mutations in dominant CTL epitopes (21), and certain LRAs impair CTL function (22–25). Therefore, curative approaches will likely require therapeutic agents that promote killing of infected cells in combination with effective LRAs.

The oncology field provides examples of targeted therapies that enhance CD8^+^ T cell responses, resulting in successful treatment of certain cancers (26). Chimeric antigen receptor (CAR)-T cell approaches have received considerable attention (26–28) but require modification and reinfusion of patient-derived effector cells. In contrast, bispecific antibody engagers that link CTLs to antigen-bearing target cells and simultaneously activate cytolytic effector function do not require ex vivo manipulation of patient cells (29). Blinatumomab, a bispecific antibody against CD3 and CD19, allows CD3-expressing T cells to kill CD19-expressing B cell precursors and improves survival in relapsed or refractory acute lymphoblastic leukemia (30). Early efforts to promote killing of HIV-1–infected cells using bifunctional molecules utilized immunotoxins targeting the HIV-1 Envelope (Env) proteins gp120 or gp41 (31, 32). More recently, bispecific antibodies against Env and CD3 (33, 34) have yielded promising results in ex vivo latency clearance assays with cells from PLWH (34). However, the low level of Env expression on infected cells (35–37) and the extraordinary sequence variation in this protein highlight the need for alternative approaches.

Here we describe an approach to target HIV-1 peptide:MHC complexes (pMHC) using TCR mimic (TCRm) bispecific antibodies capable of linking CTLs to infected target cells and promoting target cell lysis. This approach differs from related approaches based on soluble TCRs in that we used phage display to identify antibody-like reagents that bind with high affinity to HIV-1 pMHC complexes. Thus, this approach does not suffer from the limitations in affinity characteristics of TCR-based approaches. Phage screening identified single-chain variable fragments (scFvs) with much higher affinity for target pMHC (38–40). TCRm antibodies of this kind have been generated against tumor-associate peptides (38, 39) and can reduce tumor burden in an in vivo murine model (38, 39). Here, we generated novel bispecific TCRm reagents against CD3 and HIV-1 peptides bound to human leukocyte antigen (HLA) HLA-A*02:01 (A2) complexes, an allele present in >40% of the US Caucasian population (41), and evaluated their ability to mediate killing of infected cells.

## Results

### Identification of A2-Restricted CTL Epitopes Physically Presented on Infected CD4^+^ T Cells.

HIV-1 pMHC-I complexes on infected cells serve as ideal targets for antibody-based retargeting strategies. Epitopes can be identified based on predicted MHC I binding (42) and functional studies with synthetic peptides, but sequencing of peptides from purified class I molecules provides the strongest evidence that a particular p:MHC-I complex is present on infected cells. The identification of minute numbers of viral peptides within a vast excess of self-peptides has been challenging using traditional data-dependent mass spectrometry (MS), which relies on peak intensities to identify peptide hits. Therefore, we utilized a highly sensitive Poisson detection liquid-chromatography data independent acquisition MS (LC-DIAMS) method (43, 44). LC-DIAMS relies on in silico algorithms to predict putative high-affinity CTL epitopes binding to MHC alleles of interest (45). These peptides are then synthesized and their fragmentation patterns and elution positions relative to a set of retention time peptides are measured and archived. These features are used by the Poisson algorithm to identify the elution and fragmentation of low levels of the target peptide against a complex background of endogenous peptides. Identified peptides can then be quantitated in a subsequent run by adding isotope-labeled peptide analogs prior to acid elution, such that subsequent processing steps are shared. If native Western blots demonstrate no HLA in the clearing pellet and complete HLA depletion from the soluble lysate, all HLA complexes are on the beads and relative ion peak intensities between the native and labeled peptides convey relative abundances.

**Fig. 1.**
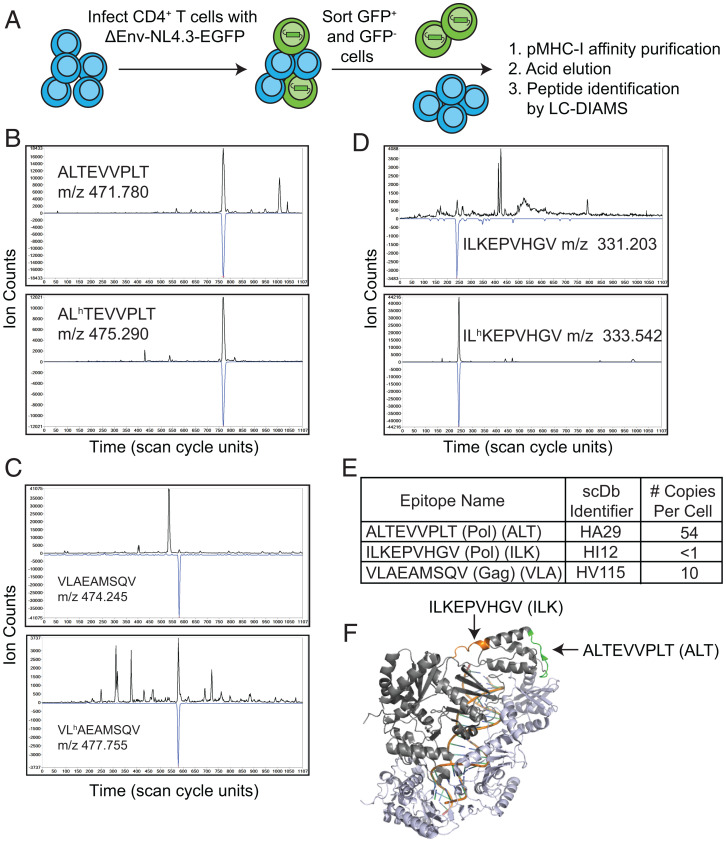
Physical identification of HLA-A*02:01–bound HIV-1 peptides by MS from infected CD4^+^ T cells. (*A*) Method for generating infected CD4^+^ T cells for physical detection of HLA-A*02:01–bound HIV-1 epitopes. (*B–D*) Detection of the native and isotope-labeled forms of HIV-1 peptides ALT, VLA, and ILK in GFP^+^ cells by targeted LC-MS/MS. Detection is indicated by elution coincidence between the extracted ion chromatogram (XIC) for the peptide’s precursor *m/z* (top traces, in black) and the Poisson chromatogram, related to the probability of the peptide’s fragmentation pattern (bottom, inverted blue trace, scaled to XIC maximum). The heavy and light forms of the peptides must also coelute, being distinguished only by different precursor masses. The relative XIC peak amplitudes, scaled by the amount of added heavy peptide, indicate the amount of the native peptide on the GFP^+^ cells. This can be converted into copies per cell (*SI Appendix*, Figs. S1–S3). (*E*) Three HLA-A*02:01 epitopes of HIV-1 are identified at different copy numbers per cell via LC-DIAMS and were the targets for generating specific scDbs (see Fig. 2). (*F*) RT epitopes are highlighted on the PBD structure 5TXM.

Across the HIV-1 proteome (excluding the variable Env protein), 107 peptides were predicted to bind to HLA-A*02:01 with high affinity and were synthesized (*SI Appendix*, Table S1). Fragmentation patterns could be identified for 64 peptides, with cysteine-containing peptides and very hydrophobic peptides explaining most of the misses. We applied LC-DIAMS to identify HLA-A*02:01–restricted epitopes presented on CD4^+^ T cells that had been infected in vitro with a GFP-tagged HIV-1 that is capable of a single-round infection (hereafter referred to as ΔEnv-NL4.3-EGFP) (46). Briefly, CD4^+^ T cells from an HLA-A2^+^ healthy donor were activated with CD3/CD28 Dynabeads and infected with ΔEnv-NL4.3-EGFP. On day 3, cells were sorted into GFP^+^ and GFP^−^ fractions and snap frozen (Fig. 1*A*). Frozen cells were lysed, and class I MHC molecules were immunoaffinity-purified with the pan-MHC I antibody W6/32. Peptides were eluted from bead-bound pMHC using low pH for subsequent LC-DIAMS analysis.

Poisson detection LC-DIAMS identified two epitopes physically present on the surface of GFP^+^ but not GFP^−^ cells: ALTEVVPLT (ALT) from reverse transcriptase (RT) and VLAEAMSQV (VLA) from the p2p7p1 region of the Gag polyprotein (Fig. 1 and *SI Appendix*, Fig. S1 *A* and *B* and Table S2). While ALT and VLA served as promising targets due to their physical identification on the surface of infected cells, we also analyzed as a positive control a previously characterized, highly conserved RT epitope ILKEPVHGV (ILK, Pol 464 to 472; RT 309 to 317), whose molecular ion we subsequently recognized in the third charged state at *m/z* 331.2 (*SI Appendix*, Table S2). To quantitate the surface presentation of these peptides, we used targeted MS/MS Poisson detection with isotope labeled leucine (L*): AL*TEVVPLT, VL*AEAMSQV and IL*KEPVHGV. ALT was identified at 54 copies (Fig. 1*B* and *SI Appendix*, Fig. S2 *A* and *B*) and VLA at 10 copies per cell (Fig. 1*C* and *SI Appendix*, Fig. S2 *C* and *D*). Surprisingly, although ILK has been identified as a target of CD8^+^ T cells from PLWH, this peptide was detected from GFP^+^ cells at a copy number of <1 per cell (Fig. 1 *D* and *E* and *SI Appendix*, Fig. S3*C*). These results are particularly striking as the epitope derives from the same viral protein as ALT (Fig. 1*F*). Calculated pMHC copy numbers per cell are an average, and certain cells may express higher or lower amounts of a given pMHC. Even nominally higher levels of ILK (i.e., one to five copies) would be nearing the limit of CD8^+^ T cell-based detection by the TCR (47, 48). Thus, we reasoned that the ILK pMHC-I target may provide insights into the limit of sensitivity of antibody-based retargeting strategies.

### A Phage-Display Library Can Be Used to Isolate Specific HIV-1 pMHC-I scFvs.

**Fig. 2.**
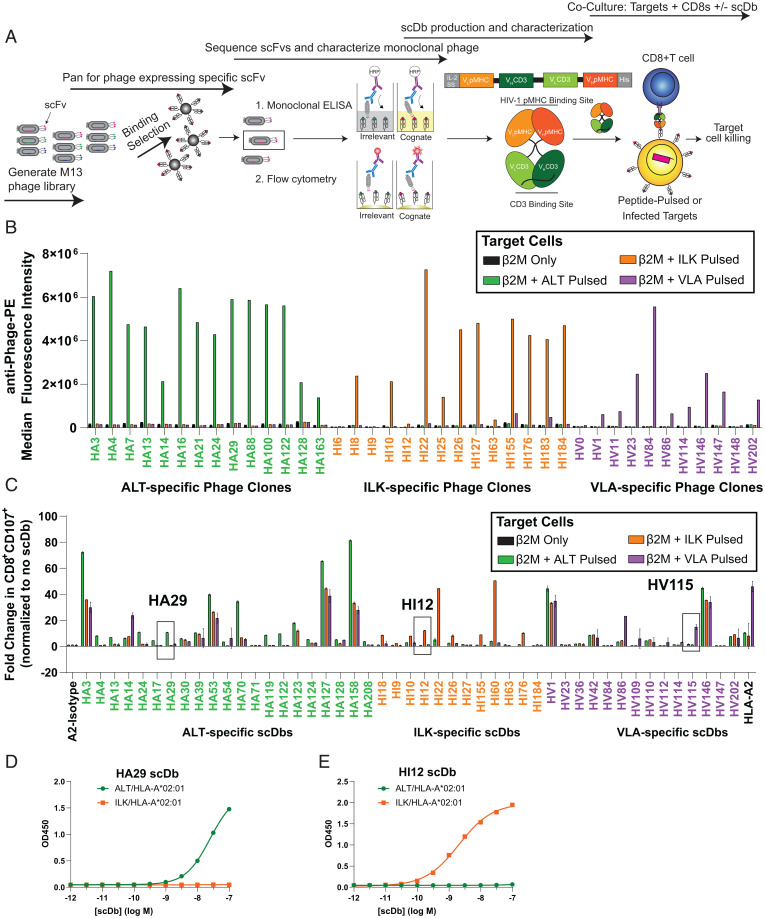
Isolation of HIV-1 pMHC-specific scFv and conversion to a scDb format. (*A*) Schematic of phage panning and characterization for the isolation of phage clones bearing scFvs specific for HIV pMHC-I. V_L_ and V_H_ sequences from the scFv of specific phage clones are cloned and expressed as scDbs. Secreted scDbs are purified by nickel chromatography and tested for functionality in cocultures with target cells (peptide-pulsed cells or infected CD4^+^ T cells) and prestimulated CD8^+^ T cells. (*B*) Monoclonal phage clones found to bind specifically to target pMHC-I by ELISA (*SI Appendix*, Fig. S4) were concentrated and tested for binding to T2 cells pulsed with relevant or irrelevant peptides. Phage specific for ALT pMHC-I (i.e., HA phage clones) bind to ALT-pulsed T2 cells (green) but not ILK-pulsed (orange) or VLA-pulsed (purple) cells. Specific binding of HI and HV monoclonal phage are also shown. (*C*) scFv from specific phage clones from *B* and *SI Appendix*, Fig. S5 *A*–*C* and *G* were cloned and expressed as scDbs against ALT (HA-scDb), ILK (HI-scDb), and VLA (HV-scDb). scDbs were screened for specific activation of CD8^+^ T cells as measured by surface CD107 when cocultured with T2 cells pulsed with relevant or irrelevant peptides. Boxes indicate the HA-, HI-, and HV-scDb exhibiting the greatest potency and specificity of CD107 activation. Data shown represent mean ± range of two biological replicates. Increasing concentrations of (*D*) HA29-scDb or (*E*) HI12-scDb binding to 1 μg/mL immobilized ALT/HLA-A*02:01 (green) or ILK/HLA-A*02:01 (orange), respectively, was assessed via ELISA. Data shown represent mean ± SD of three technical replicates.

Given the difficulty of generating antibodies that recognize pMHC complexes (49), we used a previously generated phage-display library with a diversity of ∼3.6 × 10^10^ unique clones (50, 51) to screen for scFv-bearing phage that specifically bound HLA-A*02:01 in complex with ALT, ILK, and VLA peptides (Fig. 2*A*). Phage panning to enrich for scFv-bearing phage specific to these pMHC complexes involved positive selection against decreasing amounts of the cognate pMHC and negative selection against A2-expressing cell lines and streptavidin beads bound to biotinylated HLA-A2 complexes bearing irrelevant peptides (*Materials and Methods*) (38, 39, 51). Monoclonal phage present after four rounds of panning were tested for specific binding to pMHC containing the cognate HIV-1 epitopes ALT (HA), ILK (HI), and VLA (HV), first by ELISA and then by flow cytometry. Phage scFv clones exhibiting greater than twofold binding to the cognate epitope versus an irrelevant A2 pMHC on ELISA (*SI Appendix*, Fig. S4) were amplified. Selected phage by ELISA were then screened for binding to TAP-deficient T2 cells pulsed with cognate or irrelevant peptide epitopes, and phage binding was quantified by flow cytometry (Fig. 2*B* and *SI Appendix*, Fig. S5 *A–C*). All three peptides induced stabilization of HLA-A2 on T2 cells (*SI Appendix*, Fig. S5*D*). We considered phage clones that bound to cognate versus irrelevant peptide-pulsed T2 cells at mean fluorescent intensity ratios of >4 as “hits” and determined the scFv sequences of these clones (*SI Appendix*, Fig. S5 *A–C* and *E*). Phage hits at the lower limit of the threshold were assessed for enhanced binding with different amounts of phage (*SI Appendix*, Fig. S5*F*). For the VLA/HLA-A*02:01 target, additional screening led to the identification of two specific phage clones, HV115 and HV154 (*SI Appendix*, Fig. S5*G*). These clones exhibited the greatest specificity and binding to VLA/HLA-A*02:01, but exhibited a greater background binding than either HI or HA phage clones. In total, we identified 47 specific phage clones (HA = 21, HI = 12, HV = 14).

### HIV-1 pMHC-I scFvs Can Be Converted to Single-Chain Diabodies that Induce Specific T Cell Activation.

The scFv sequences of phage hits were converted into a single-chain diabody (scDb) format (38, 39) consisting of a single polypeptide chain containing the heavy- and light-chain variable regions of two scFv fragments separated by flexible glycine repeat linkers (Fig. 2*A*). The scFv of the anti-pMHC domain derived from phage panning flanks the scFv of the UCHT-1 clone of CD3ε (38). Binding of the scDb to CD3 on the effector cell and the HIV-1 pMHC on the infected target cell tethers the effector to the target to form an immune cytolytic synapse, with activation of the effector cell as assessed by CD69 and CD107 up-regulation, release of cytokines and chemokines, such as interferon (IFN)-γ and MIP1β (38, 39), and target cell lysis.

To test the functionality of the scDbs, we cocultured CD8^+^ T cells from healthy donors with T2 cells pulsed with cognate or irrelevant peptides and assessed effector cell activation by cell surface expression of CD107 (52) (Fig. 2*C*). Some scDbs were inactive or nonspecific (Fig. 2*C*). The most specific phage clones against ALT were HA122 and HA29 (Fig. 2*C*). The most specific ILK clones were HI12 and HI55, and the most specific VLA clone was HV115 (Fig. 2*C*). HA29 and HI12 induced higher levels of CTL activation, as measured by secreted MIP1β, compared to HI55 and HA122 (*SI Appendix*, Fig. S6*A*). These two scDbs also induced enhanced MIP1β production by and CD69 expression on CD8^+^ T cells in response to cells pulsed with lower doses of cognate peptide compared with the other top scDbs HA122, HI55, or HV115 (*SI Appendix*, Fig. S6 *B–D*).

Additional testing of HA29- and HI12-scDbs using titration ELISAs highlighted their specificity. The HA29-scDb only bound to immobilized ALT/HLA-A*02:01 and not the irrelevant monomer ILK/HLA-A*02:01, even at high concentrations of HA29-scDb (Fig. 2*D*). Similarly, HI12 bound to only ILK/HLA-A*02:01 and not the irrelevant target ALT/HLA-A*02:01 (Fig. 2*E*). The specificity of HA29-scDb was additionally interrogated using positional scanning variant peptides, in which each residue of the ALT peptide was substituted with 19 other possible amino acids (38, 39). This resulted in a library of 171 variant peptides. HA29-induced up-regulation of CTL activation was significantly decreased in cocultures with T2 cells pulsed with peptide variants containing substitutions in positions 3 to 5 (*SI Appendix*, Fig. S7), suggesting that these residues were most critical for HA29 binding. Positions 1 and 7 tolerated more substitutions but also were critical for scDb binding (*SI Appendix*, Fig. S7), while substitutions at positions 6, 8, and 9 in many cases did not significantly affect HA29 binding. Due to their superior activity and specificity, we pursued additional characterization of the scDbs HA29 and HI12.

### HIV-Specific scDbs Have High Affinity and Sensitivity for Their Cognate pMHC.

**Fig. 3.**
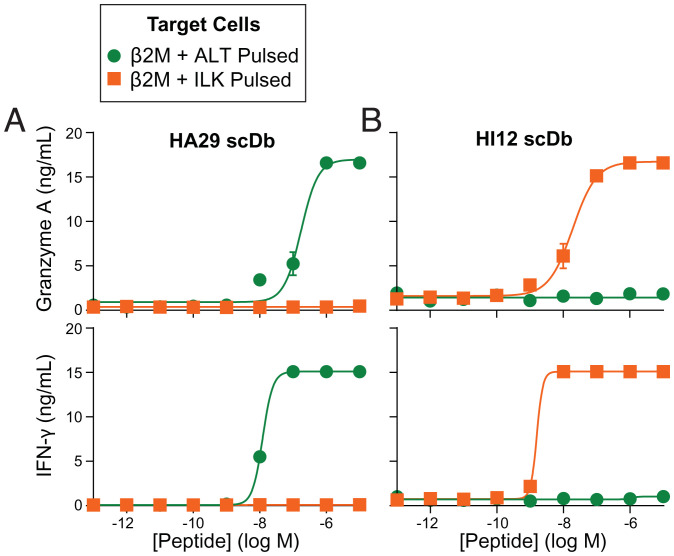
HA29- and HI12-scDbs induce polyfunctional CD8^+^ T cell responses. T2 cells were pulsed with the ALT or ILK peptides at the indicated concentrations. Next, 10 × 10^4^ peptide-pulsed cells were cocultured with 5 × 10^4^ preactivated CD8^+^T cells (1:2 E:T) in the presence of 0.25 nM HA29- or HI12-scDb for 72 h. Supernatants were assayed with Legendplex or MIP1β ELISAs. Specific activation of CD8^+^T cells by (*A*) HA29 or (*B*) HI12 was observed by the secretion of Granzyme A (*A* and *B*, *Top*) and IFN-γ (*A* and *B*, *Bottom*) in response to cells pulsed with the cognate but not irrelevant peptide. Data indicate mean ± range of two biological replicates. Similar specific responses were also observed using assays for other effector molecules (*SI Appendix*, Fig. S8).

For TCRm reagents to promote killing of infected cells, they must detect viral peptide-MHC I complexes presented at low copies on target cells. We performed peptide titration cocultures of T2 cells and healthy donor-derived CD8^+^ T cells in the presence or absence of scDbs. At 0.25 nM, both HA29 and HI12 induced specific, polyfunctional T cell responses against cells pulsed with nanomolar concentrations of the relevant peptide (Fig. 3 *A* and *B* and *SI Appendix*, Fig. S8). In addition to inducing canonical markers of CTL degranulation (granzyme A, Fig. 3* A* and *B*, *Top*) and cytokine release (IFN-γ) (Fig. 3 *A* and *B*, *Bottom*), we demonstrated specific induction of release of multiple additional granule proteins and effector molecules, cytokines, and chemokines, including granzyme B, granulysin, perforin, sFasL, tumor necrosis factor (TNF)-α, interleukin (IL)-2, IL-4, IL-17A, and MIP1β (*SI Appendix*, Fig. S8).

**Fig. 4.**
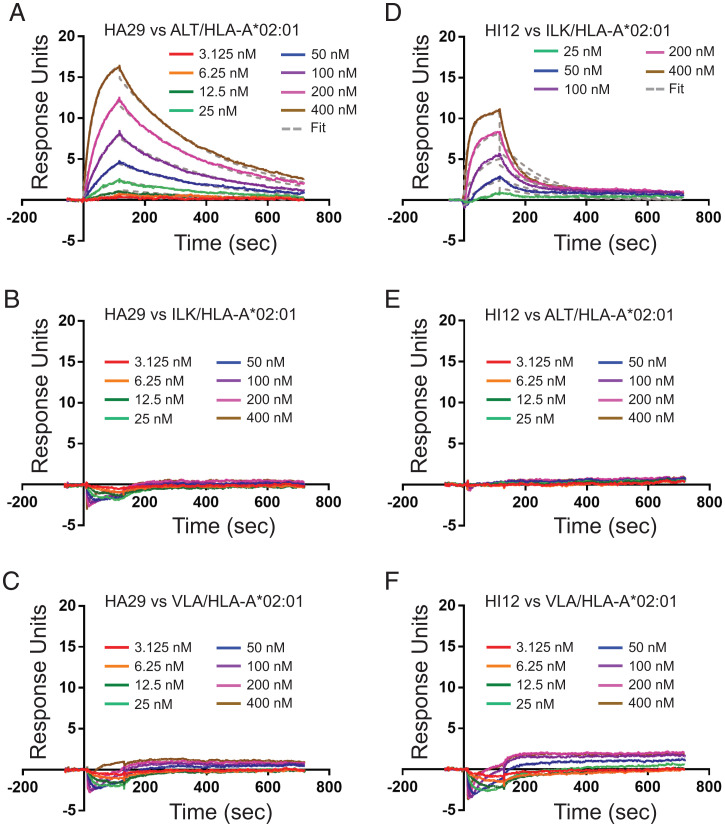
HIV-specific scDbs have high affinity for their cognate pMHC-I. (*A*) HA29-scDb binding to ALT/HLA-A*02:01 was measured with multicycle kinetics using SPR at concentrations up to 400 nM. (*B* and *C*) No binding was observed with HA29 against irrelevant epitopes ILK/HLA-A*02:01 and VLA/HLA-A*02:01 at all concentrations tested. (*D*) HI12-scDb binding to ILK/HLA-A*02:01 was measured with multicycle kinetics using SPR. (*E* and *F*) No binding was observed with ILK against irrelevant epitopes ALT/HLA-A*02:01 and VLA/HLA-A*02:01 at all concentrations tested. All SPR measurements were done in duplicate.

Surface plasmon resonance (SPR) analysis demonstrated HA29 binding to ALT/HLA-A*02:01 with an equilibrium constant (*K*_D_) of 53.8 nM (Fig. 4*A*), an association rate constant (*k*_on_) of 5.98 × 10^4^ M^−1^s^−1^, and a dissociation rate constant (*k*_off_) of 3.22 × 10^−3^ M^−1^s^−1^. No binding of the HA29-scDb to ILK pHLA-A2 or VLA pHLA-A2 was detected (Fig. 4 *B* and *C*). Similar analysis of HI12 demonstrated binding to ILK/HLA-A*02:01 with a *K*_D_ of 66.8 nM (Fig. 4*D*), a *k*_on_ of 9.48 × 10^4^ M^−1^s^−1^, and a *k*_off_ of 6.58 × 10^−3^ M^−1^s^−1^. No binding of the HI12-scDb to ALT pHLA-A2 or VLA pHLA-A2 was detected (Fig. 4 *E* and *F*). These data are consistent with the high potency and specificity of the HA29- and HI12-scDbs (Fig. 3), suggesting that these scDbs may detect pMHC-I on infected cells and induce killing.

### HA29 and HI12 Induce Robust and Polyfunctional CTL Activation against Peptide-Pulsed Cells.

**Fig. 5.**
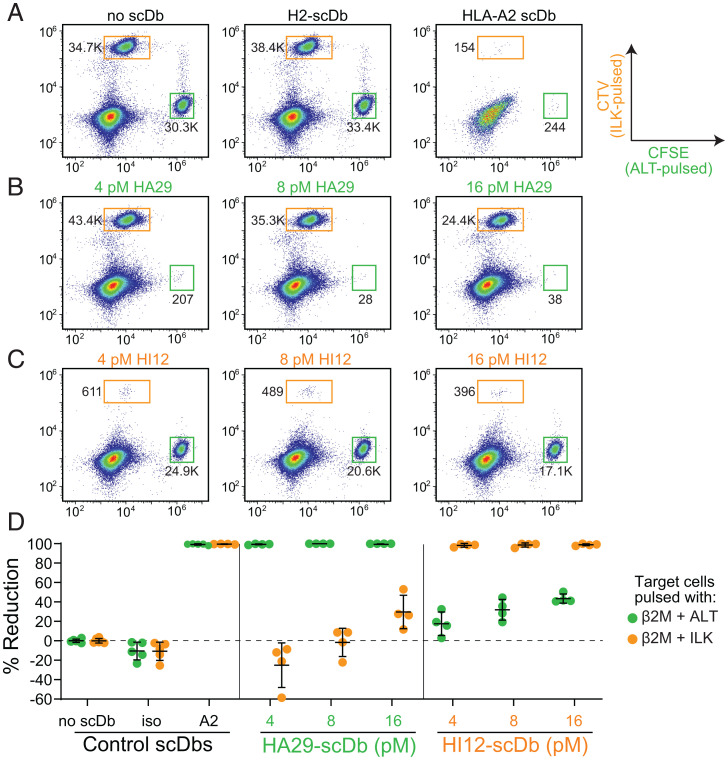
HA29- and HI12-scDbs induce killing of target cells bearing relevant pMHC-I. T2 cells were pulsed with ALT or ILK peptides and β2M and stained with CFSE or CTV, respectively. Next, 15 × 10^4^ preactivated CD8^+^T cells were cultured with 5 × 10^4^ peptide-pulsed, CFSE- and CTV-stained cells (3:1:1 E:T) in the presence of HA29- or HI12-scDbs for 18 h and assayed for specific killing. Representative flow-cytometry plots showing the frequency of the ALT-pulsed (CFSE^hi^) and ILK-pulsed (CTV^hi^) populations in cocultures with (*A*) no scDb, 250 pM of isotype H2-scDb, and 250 pM of pan-A2-scDb; (*B*) increasing doses of the HA29-scDb; or (*C*) increasing doses of HI12-scDb. Flow plots were gated on lymphocyte size, single cells, and viable cells. Numbers by the gates indicate bead-corrected cell counts. (*D*) Percent reduction of ALT-pulsed (CFSE^hi^) or ILK-pulsed (CTV^hi^) populations mediated by HA29- or HI12-scDbs. Data represent the mean ± SD of four biological replicates.

To test the ability of HA29 and HI12 to induce cytolysis of target cells bearing the relevant pMHC, T2 cells pulsed with the ALT or ILK peptides were labeled with 5-(and 6)-Carboxyfluorescein diacetate succinimidyl ester (CFSE) or cell-trace violet (CTV), respectively, and cocultured with preactivated CD8^+^ T cells in the presence or absence of increasing doses of scDb. For negative controls, we used a no-scDb condition (Fig. 5 *A*, *Left*) and an isotype scDb (H2) directed against a mutant p53 epitope presented on HLA-A*02:01 (Fig. 5 *A*, *Center*) (39). We used a pan HLA-A2 (BB7.2) scDb as a positive control to identify the maximum killing of target populations (Fig. 5 *A*, *Right*). At picomolar concentrations, HA29- and HI12-scDbs induced nearly 100% reduction in T2 cells pulsed with the relevant peptide (Fig. 5 *B* and *C*), similar to the reductions obtained with 250 pM of the positive-control pan-A2 scDb (Fig. 5*D*). At the lowest concentrations of HA29 and HI12 (4 pM), there was minimal effect on targets pulsed with the irrelevant peptide.

### Suppression of HIV-1 Using HA29 but Not HI-12 scDbs.

**Fig. 6.**
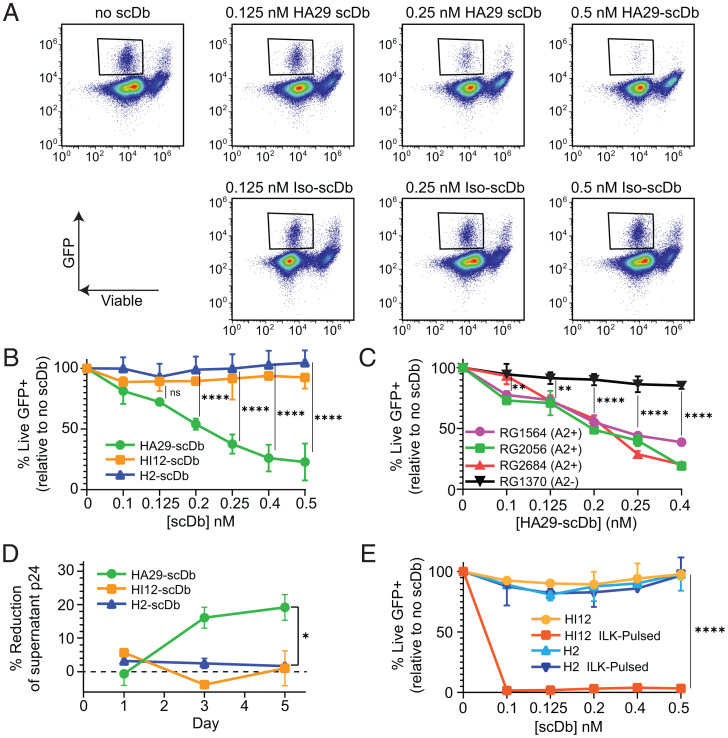
HA29-scDb induces dose-dependent viral suppression. (*A*) Representative flow plots from a suppression assay showing the dramatic decrease in viable GFP^+^ cells with increasing concentrations of HA29-scDb compared to cocultures containing isotype (H2) scDb. Activated CD4^+^ T cells from an A2-expressing healthy donor were infected with ΔEnv-NL4.3-EGFP and cocultured with autologous prestimulated CD8^+^T cells for 72 h before flow cytometric analysis (*Materials and Methods*). (*B*) Percent of live GFP^+^ cells remaining after 72 h of coculture with HA29-, HI12-, or H2-scDb and autologous CD8^+^ T cells. Results are expressed as a percent of the live GFP^+^ cells remaining in cultures without scDbs. (*C*) Suppression assays using cells from three A2^+^ and one A2^−^ donor. Results are expressed as percent of the live GFP^+^ cells remaining relative to cultures without scDbs. (*D*) Suppression of a replication-competent reservoir isolate 33A10 (56). Healthy donor CD4^+^ T cells were activated, infected with 33A10, and cocultured with autologous CD8^+^ T cells in in the presence of 0.25 nM of the HA29-, HI12-, or H2-scDb. Supernatant p24 was measured by ELISA after 1, 3, and 5 d of coculture. Reduction in p24 was normalized to the no scDb control. (*E*) Target cells (infected cells or infected cells pulsed with 10 μg/mL ILK peptide) were cocultured with autologous CD8^+^ T cells in the presence of the HI12- or H2-scDbs. Results are expressed as a percent of the live GFP^+^ cells remaining in cultures without scDbs. Data in represent the mean ± SD of three independent experiments analyzed by two-way ANOVA followed by Tukey’s multiple comparison test. Data in *C* represent the mean ± SD of four independent experiments analyzed by two-way ANOVA followed by Tukey’s multiple comparison test. Data in *D* and *E* represent the mean and range of two technical replicates and were analyzed by two-way ANOVA followed by Tukey’s multiple comparison test. *P < 0.05, **P < 0.01, ***P < 0.001, ****P < 0.0001.

Given the HA29- and HI12-scDb induced killing of peptide-pulsed cells, we tested whether scDbs could inhibit viral replication or induce direct lysis of infected cells using viral suppression assays (53). On infected cells, the level of specific peptide-MHC complexes is extremely low (54 copies of ALT/HLA-A*02:01 per cell) (*SI Appendix*, Fig. S2 *A* and *B*), too low to be detected by flow cytometry. Nevertheless, we hypothesized that the extremely sensitive nature of antigen recognition by T cells might allow scDb-directed lysis of infected cells. CD4^+^ T cells from HLA-A2–expressing healthy donors were activated, infected with ΔEnv-NL4.3-EGFP (46), and cocultured with autologous, prestimulated CD8^+^ T cells in the presence of HA29- or HI12-scDb at an effector-to-target (E:T) ratio of 3:1. We observed a striking dose-dependent decrease in viable GFP^+^ cells remaining after 3 d of coculture with the HA29-scDb, but no decrease with the HI12 or irrelevant H2-scDb (Fig. 6 *A* and *B*). At 0.4 nM HA29-scDb, the number of viable GFP^+^ cells was only 24% of that seen without scDb (Fig. 6*B*). The decrease in residual GFP^+^ cells mediated by HA29-scDb was dependent on HLA-A2, as no significant reductions in viable GFP^+^ cells were observed with an A2^−^ donor (Fig. 6*C*). HIV-1 Nef and Vpu down-regulate MHC-I (54, 55), and we observed decreases in HLA-A2 on infected CD4^+^ T cells (*SI Appendix*, Fig. S9*A*). Despite lower A2 on infected cells, we observed suppression by HA29-scDb (*SI Appendix*, Fig. S9*B*), suggesting that the residual numbers of viral pMHC-I were sufficient and the affinity of HA29-scDb was high enough to maintain a suppressive effect. Indeed, we observed HA29-mediated CTL activation even at low doses of the HA29-scDb in infected versus uninfected cocultures (*SI Appendix*, Fig. S9*C*). Importantly, we did not observe a significant decrease in viability in uninfected CD4^+^ T cells cocultured with autologous CD8^+^ T cells in the presence of HA29-scDb (*SI Appendix*, Fig. S9*D*).

Having shown viral suppression using a standard HIV-1 reporter virus, we next confirmed that HA29-scDb could suppress a replication-competent primary isolate from the latent reservoir of a treated PLWH. We infected primary CD4^+^ T cells with isolate 33A10 (56) and measured p24 production over time with and without scDbs in the presence of autologous CD8^+^ T cells. Levels of p24 antigen were reduced by the HA29-scDb (Fig. 6*D*). Together, these results demonstrate that the HA29-scDb can induce CD8^+^ T cell-mediated killing of HIV-1–infected cells. This is dependent on the presence of A2 and antigen. Interestingly, as with ΔEnv-NL4.3-EGFP–infected cells (Fig. 6*B*), we did not observe a suppressive effect with the HI12-scDb, despite its high sensitivity for detecting T2 cells pulsed with nanomolar concentrations of peptide (Figs. 3*B* and 6*B* and *SI Appendix*, Fig. S8*B*) and its high affinity for ILK/HLA-A*02:01 (Fig. 4*D*). The inability of the HI12-scDb to mediate killing may be due to lower levels of ILK pMHC-I on infected cells, as suggested by quantification of the ILK peptide from GFP^+^ cells by MS (*SI Appendix*, Fig. S3 *A* and *B*). Indeed, when we pulsed infected CD4^+^ T cells with saturating a concentration (10 μg/mL) of the ILK peptide, we observed a nearly 100% decrease in the residual viable GFP^+^ population compared to the irrelevant H2-scDb (Fig. 6*E*). Therefore, antigen density on HIV-1–infected cells may limit the effectiveness even of even high-affinity scDbs.

## Discussion

The importance of a robust CD8^+^ T cell response in HIV-1 infection is highlighted by studies of elite suppressors (ES), who suppress viremia to undetectable levels without cART (57, 58). The magnitude and characteristics of the CD8^+^ T cell response in ES (19, 59, 60), and the targeting of highly networked epitopes (61), have been implicated in their ability to control viral replication. These studies have led to efforts to stimulate CTLs in PLWH using therapeutic vaccination strategies for cure efforts. This has proven challenging, however, as HIV-1–specific CTLs in chronic progressors have a hypofunctional phenotype that is not completely restored by cART (18, 19, 62, 63). HIV-1–specific CTLs also require prestimulation in order to clear infected cells after latency reversal (20). Retargeting strategies, such as the pMHC-I scDb strategy outlined above, are beneficial in that they enable CD8^+^ T cells of any specificity to recognize HIV-infected cells, thereby bypassing defects unique to the HIV-1–specific CTL subpopulation.

Here we describe bispecific antibodies against HIV-1 pMHC-I expressed on infected cells. Bispecific antibodies capable of detecting rare HIV-1 pMHC complexes on infected cells might be useful in eradicating the latent reservoir following latency reversal. To identify target peptides, we used a highly sensitive MS method to quantitate HIV-1 peptides presented on MHC-I isolated from infected cells. Phage-bearing scFv specific for the identified pMHC-I were then isolated by panning from an extremely diverse library and cloned into an scDb backbone.

Two scDbs, HA29 and HI12, exhibited high affinity and specificity for their respective pMHC targets. Subnanomolar concentrations of both scDbs induced polyfunctional CTL activation against T2 cells pulsed with peptides in the 0.1 to 10 nM range. Prior studies involving single-molecule immunofluorescence using an affinity-matured TCR against a melanoma pMHC suggest that peptide concentrations of 0.1 to 10 nM should result in the presentation of physiological levels of pMHC (4 to 80 copies per cell) (64). Since the HA29 and HI12 bispecific antibodies enabled CD8^+^ T cell recognition of cells pulsed with 0.1 nM peptide, we predicted these antibodies could detect endogenously processed epitopes on infected cells. When HA29- and HI12-scDbs were incubated with CTLs and newly infected CD4^+^ T cells, HA29-scDb suppressed viral infection in a 72-h coculture. In contrast, the HI12-scDb did not, consistent with the lower copy number of the ILK epitope as quantitated by MS (greater than one copy of ILK/HLA-A*02:01 per cell). Pulsing infected cells with the ILK peptide and then coculturing them with CTLs led to dramatic suppression/killing, implicating low antigen density as the primary factor preventing HI12-induced killing.

Our results are consistent with prior studies. Tsomides et al. (65) generated a stable, HLA-A2^+^ Jurkat cell line carrying a *nef*-deficient HIV-1 provirus and estimated the ILK pMHC density at ∼10 copies per cell. An ILK-specific T cell clone required very high (i.e., 50:1) E:T ratios to promote killing of the cell line (65). If the density of ILK on cells constitutively expressing HIV-1 protein and A2 levels is only ∼10 copies (66), it follows that in the setting of natural infection, presentation of ILK on the cell surface would be even lower. ILK is derived from the RT protein, which is expressed at only 10% of the level of the more abundant Gag protein due to a ribosomal frame shift required for RT translation, but this alone is not sufficient to explain the low levels of ILK. The ALT peptide, found at 54 copies per cell, derives from the same protein. Thus, the difference between the RT ALT and ILK copy numbers (54 vs. <1 per cell) may reflect differences in processing. Although the two epitopes are located within 12 residues of each other, ALT (amino acids 290 to 298) is located on an extruded loop of RT and contains one residue of an α-helix that precedes ILK (amino acids 309 to 317). ILK is located at the C-terminal end of the same constrained α-helix that separates these two epitopes. The entire 17-amino acid helix is likely less easily unfolded, and thus may impact translocation through the proteosome or protein cleavage to diminish the generation of the downstream ILK epitope (67). Importantly, other factors may also contribute to the efficacy of the scDb approach describe here. In addition to peptide affinity for the relevant MHC molecule, these include the effect of flanking residues on cleavage by the proteasome and also other amino peptidases, such as ERAP1 and ERAP2, TAP transport, association with molecular chaperones, and stability of the complexes.

Our results highlight the need to reexamine the optimal CTL epitopes for viral control in the context of antigen density (68). Most studies have analyzed the conservation of CTL epitopes (21, 69, 70). Recently, network analysis has identified epitopes that are mutationally constrained (61). The consensus is that targeting highly conserved or mutationally constrained epitopes would be therapeutically beneficial. However, highly conserved epitopes, such as ILK, may not be presented at sufficient levels to serve as appropriate targets. Responses to the ILK epitope were first observed using T cell clones from asymptomatic HIV^+^ individuals that lysed autologous cells infected with vaccinia vectors expressing HIV-1 genes (71, 72). However, vaccinia infection may produce proportionally more viral pMHC than would occur from natural HIV-1 infection (65).

Many cure strategies aim to target HIV-1 Env on the surface of infected cells (73). However, Env has an extremely high sequence diversity (74). Broadly neutralizing antibodies (bNAbs) address this issue (75) and have been reformatted to include anti-CD3 domains to engage T cells (33, 34). However, anti-Env bNAbs can drive selective pressure (76) and the cell-surface levels of HIV-1 Env are low on infected cells (36, 37). Anti-pMHC scDbs could synergize with anti-Env scDbs to enhance CTL killing of infected cells following latency-reversal, providing a complementary arm for the “kill” in shock-and-kill efforts.

There are several potential benefits to using pMHC-specific scDbs in the situation where latency has been successfully reversed in the context of a shock-and-kill strategy. The large diversity of targets against which scDbs can be generated throughout the viral proteome provides a level of versatility that anti-Env bNAbs do not offer. A “cocktail” of bispecific engagers—targeting conserved pMHC presented at relatively high copy numbers from various viral proteins—could trigger CTL-mediated lysis of infected cells in a manner that would preclude viral evasion from any one scDb. Although the scDbs described here showed high affinity and specificity for the target pMHC, higher concentrations of the scDbs exhibited some nonspecific activity against irrelevant targets. Higher affinity and specificity could be achieved by additional scFv selection using affinity-matured libraries. The same methodology can be extended to other HLA alleles to increase the population coverage. SIV antigens in infected cells may be processed and presented using alternative pathways on MHC-II and HLA-E (77–79). As HLA-E is nearly monomorphic, targeting a conserved HIV-1 peptide bound to HLA-E using the above approach may provide a nearly universal bispecific reagent. Thus, the strategies described here could be used to isolate scFv specific for nonclassically presented HIV-1 peptides. Another benefit of scDb is that they induce CTL activation even at low antigen densities, as seen from our peptide-pulsing experiments. Given the variability in LRA-mediated reactivation of latent HIV-1 (56, 80), repeated infusions of scDbs might be required to reduce the latent reservoir.

Whether scDb are able to activate CTLs better than natural recognition by αβTCRs remains to be seen. Contrary to conventional receptor–ligand interactions exemplified by antigen–antibody binding, bioforces are essential for nonthermal equilibrium, mechanosensor-based αβT cell activation (81–90). αβT cell motility during immune surveillance and the local cytoskeletal machinery place physical load on individual αβTCR-pMHC bonds, tuning the sensitivity and specificity of αβTCR recognition (82). In the absence of external load, chemical thresholds to trigger cellular αβT cell activation require a 1,000-fold or higher number of pMHC molecules than observed physiologically (83). In contrast, under force the ligand-mediated induction of αβT cell biological response can be essentially digital (i.e., single pMHC recognition). That said, it remains to be seen how soluble bispecific reagents versus digitally performing αβTCRs used for adoptive cellular immunotherapy compare head-to-head in detection of the same pMHC ligand on target cells to effectuate specific killing. Clustering of bispecific reagents, perhaps on nanoparticle arrays, could potentially increase sensitivity.

There are potential limitations to the use of bispecific antibodies to eliminate HIV-1–infected cells in HIV-1 cure strategies. It is possible that the bispecific antibodies will be immunogenic and induce antidrug antibodies that will limit their effectiveness. The bispecifics described here were generated using scFv phage-display libraries based on a humanized antibody (38, 39). The extent to which the development of antidrug antibodies will limit the effectiveness of this approach will likely become apparent when these approaches, which are being rapidly developed for cancer immunotherapy, are more fully evaluated in clinical trials. It is also important to emphasize that the bispecific reagents described here can only eliminate infected cells that are actively expressing HIV-1 genes and producing the relevant HIV-1 proteins. These reagents have no ability to reverse HIV-1 latency and can only promote the elimination of latently infected cells if HIV-1 gene expression is first induced by effective LRAs. The discovery of LRAs that induce viral gene expressing in a large fraction of reservoir cells is a problem that has not yet been solved despite extensive effort in many laboratories (12, 14–18, 91). Many of the LRAs identified to date using model systems fail to reverse latency in ex vivo studies with resting CD4^+^ T cells from treated PLWH (80). Therefore, a successful cure strategy will likely require the discovery of more effective LRAs that will complement the killing strategy described here. In addition, the persistence of HIV-1 in macrophages and in T cells subsets that are localized to tissues sites not readily penetrated by CTL (such as germinal centers) may pose additional barriers to the elimination of infected cells (92, 93). Furthermore, reservoir cells may have been selected for intrinsic resistance to CTL killing (94). It is also important to note that many reservoir viruses contain escape mutations in immunodominant CTL epitopes (21). Fortunately, mutations are generally absent in subdominant epitopes, allowing targeting of infected cells (21). Finally, CTL dysfunction associated with the expression of PD1 may persist in the setting of ART (95). However, the exhausted phenotype is found on HIV-1–specific CTL and to a much less extent on CTL specific for other antigens. An advantage of the strategy described here is that it enables CTLs of any specificity to kill HIV-1–infected target cells.

The complications described above may contribute to the finding that shock-and-kill strategies have yet to produce substantial decrease in the latent reservoir in animal models (91). However, there is evidence for a delay in viral rebound after ART interruption in simian immunodeficiency virus-infected macaques that received both an immune stimulant with potential LRA activity (TLR7 agonist) coupled with immunologic interventions (bNAbs or therapeutic vaccination) (96, 97). The small signal observed in these studies provides hope that cure may be possible with better LRAs and more effective killing strategies, possibly including the one described here.

To our knowledge, no other studies have described the generation and use of TCRm-antibodies against HIV pMHC to target latent HIV infection. We have identified naturally processed targets on infected cells and generated reagents using phage display that could recognize and kill infected cells. Our studies highlight the importance of antigen density in the design of therapeutic vaccines and immunotherapies, and lay the groundwork for future studies that aim to eliminate the latent reservoir using bispecific cell engagers.

## Materials and Methods

### Human Samples.

Leukapheresis samples from HLA-A*02:01^+^ donors were obtained from Stem Cell Technologies. Peripheral blood mononuclear cells (PBMCs) were purified by density gradient centrifugation with Ficoll Paque Plus (GE Healthcare) and cryopreserved.

### Cell Lines and Primary Cells.

All cells were grown at 37 °C under 5% CO_2_. PBMCs were thawed in RPMI 1640 with 10% FBS, 1% penicillin/streptomycin, and rested overnight before stimulation with 15 ng/mL of anti-human CD3 antibody (clone OKT3, BioLegend) for 3 d in base media (RPMI 1640 with 10% FBS, 1% penicillin/streptomycin) containing 250 U IL-2 and 5 ng/mL IL-7 (hereafter termed IL-2/IL-7 media). OKT3 was removed after 3 d of activation and cells were maintained in IL-2/7 media at 1 × 10^6^ cells/mL for 7 to 10 d before isolation of CD8^+^ T cells by negative selection (StemCell) for coculture experiments. Separately, autologous PBMCs were also used to isolate CD4^+^ T cells by negative selection (StemCell). CD4^+^ T cells were activated using Human T-Activator CD3/CD28 Dynabeads (Thermo Fisher Scientific) in RPMI 1640 with Glutamax, 10% FBS, and 1% penicillin/streptomycin and 30 U IL-2.

T2 cells were cultured in RPMI 1640 + Glutamax with 10% FBS, 1% penicillin/streptomycin. RPMI 6666 cells were grown in the same media and used as an additional A2^+^ cell line for negative-selection stages of phage panning.

### LC-DIAMS.

LC-DIAMS requires fragmentation patterns and relative elution positions for all synthetic peptides, which are predicted to bind to HLA-A*02:01 (*SI Appendix*, Table S1). The pattern and elution map for each synthetic peptide can then be interrogated in complex samples containing minute quantities of the target peptide. For elution mapping, synthetic peptides were added to an extract of peptides from HeLa cells. Separately, peptides from HeLa digests were spiked into a sample containing A2-bound peptides eluted from W6/32 immunoprecipitation of CaSki, an HLA-A*02:01–expressing cell line. Both samples were run on LC-DIAMS. The relative position of synthetic peptides compared to HeLa peptides, and the relative position of HeLa peptides compared to endogenous A2*01-bound peptides, could be integrated to predict the elution position of synthetic HIV-1 epitopes among a background of endogenous A2*01 epitopes. These elution maps could be used to analyze whether low levels of candidate peptides were indeed presented on the surface of infected cells.

To prepare infected cells for LC-DIAMS, CD4^+^ T cells from an HLA-A2^+^ healthy donor were activated with CD3/CD28 Dynabeads and infected with ΔEnv-NL4.3-EGFP. Three days after infection, cells were sorted into GFP^+^ and GFP^−^ fractions and snap frozen. Frozen cells were lysed. MHC class I molecules were immunoaffinity purified with W6/32, and peptides were eluted from bead-bound pMHC-I using low pH. Peptide quantification of native pMHC by targeted Poisson detection was achieved by adding 100 attomoles of heavy (isotope-labeled) versions of ALT and VLA and 200 attomoles of heavy ILK to the GFP^+^ samples, as previously described (43, 44).

### Peptides and pHLAs.

Peptides were synthesized at >95% purity (Elim Biopharm or JPT Peptide Technologies) with the exception of the crude peptides used for the positional scanning library and for LC-DIAMS. Peptides were resuspended in dimethylformamide (DMF) and stored at −80 °C. HLA-A2 was refolded with peptide and β-2 microglobulin (β2M), purified by gel filtration, and biotinylated (Fre Hutchinson Immune Monitoring Lab, Seattle, WA or Baylor MHC Tetramer Production Lab, Houston, TX). Confirmation of pMHC refolding before phage panning was done using ELISA with the antibody W6/32 (BioLegend).

### Phage-Display Library Construction.

The scFv-bearing library used for phage panning was previously described (38, 39, 51) and was regrown within a week of selection.

### Phage Panning and Characterization and scDb Preparation.

Phage panning, characterization, and generation of scDbs were performed as previously described (38). See *SI Appendix* for details.

### SPR Affinity Measurements.

SPR experiments were carried out on a Biacore T200 (Cytiva) at 25 °C of an SA chip. HBS-P (10 mM Hepes pH 7.4, 150 mM NaCl, 0.05% [vol/vol] surfactant P20) was used as the immobilization and capture running buffer. The three HLA ligands used were biotinylated A*02:01 bound to ILK, ALT, and VLA peptides. Approximately ∼30 RU of each pMHC ligand was captured onto flow cells FCs 2 through 4 while flow cell FC1 was used as the reference subtraction. Bispecifics HA29 and HI12 were flown over as analytes at a rate of 50 μL/min in increasing concentration 3.125, 6.25, 12.5, 25, 50, 100, 200, and 400 nM, twofold dilutions). The scDb HI12 was flowed over FC2 (biotinylated HLA-A*02:01 bound to ILK) by increasing concentrations of 25, 50, 100, 200, and 400 nM (twofold dilutions). Multicycle kinetics were performed in the presence of HBS-P supplemented with 5% glycerol with contact and dissociation times of 120 s and 600 s, respectively. One 20-s injection of 2 M NaCl was used for surface regeneration. Binding responses for kinetic analyses were referenced and blank-subtracted. All curves were fit with a 1:1 kinetic binding model using Biacore Insight Evaluation Software. All SPR measurements were done in duplicates.

### pMHC ELISA Binding Assay.

Monomers (1 μg/mL) in BAE blocking buffer (phosphate-buffered saline or PBS, 0.5% BSA, 0.1% sodium azide) were added to a EvenCoat streptavidin-coated plate (R&D Systems) and incubated at 4 °C for 16 h. The plate was washed with 1XTBS-T (1× TBS + 0.05% Tween-20) using a BioTek 405 plate washer and scDbs (5 μg/mL in BAE) were plated and incubated at room temperature for 1 h. The plate was washed and HRP anti-6× His tag mIgG secondary (clone J099B12, BioLegend, 0.5 μg/mL in BAE) was plated and incubated at room temperature for 1 h. The plate was washed and developed in TMB for 5 min at room temperature before stopping in 1 N sulfuric acid. OD_450_ was read; all conditions were tested in triplicate, average and SD shown. For measuring the limit of scDb binding, binding was conducted as above with decreasing concentrations of scDb, as indicated.

### Peptide Titration Cocultures.

T2 cells were pulsed overnight with varying concentrations of peptide (see figures) in RPMI media containing 10 μg/mL β2M and 1% penicillin/streptomycin. Peptide-pulsed T2 cells were cocultured with preactivated healthy donor CD8^+^ T cells at a 1:2 E:T ratio in 10% FBS 1% penicillin/streptomycin RPMI in a 96-well V-bottom plate. All scDbs were added to a concentration of 0.25 nM. Supernatants were collected after 3 d of coculture. Human CCL4/MIP-1B Quantikine ELISA (R&D Systems) and Human CD8/NK Panel Legendplex (BioLegend) were performed as per the manufacturers’ protocols.

### Positional Scanning Variant Testing.

The 171 positional scanning variant peptides were obtained by substituting each residue of the original peptide with the 19 other possible amino acids, as previously described (38, 39). T2 cells were pulsed with 10 μg/mL β2M and each variant peptide at 10 μM in serum-free RPMI for 4 h. Pulsed cells were cocultured 1:1 with preactivated CD8^+^ T cells from healthy donors and 0.25 nM HA29-scDb. Coculture supernatants were assayed for MIP1β (Human CCL4/MIP-1B Quantikine ELISA, R&D Systems) at 24 h. HLA-A2 levels on pulsed cells were also assessed by surface staining with an anti–HLA-A2 antibody (BioLegend) and viability dye (eFluor 780, ThermoFisher).

### CFSE and CTV Dual Stain Coculture.

T2 cells were pulsed with 1 μg/mL peptide and 10 μg/mL β2M in serum-free RPMI for 4 h at 37 °C. ALT- or ILK-pulsed cells were counted, pelleted, and stained with CTV (ThermoFisher) or CFSE (ThermoFisher), respectively, as per the manufacturer’s instructions. Stained cells were washed three times with excess PBS; 15 × 10^4^ preactivated CD8^+^T cells were then combined with 5 × 10^4^ cells from each target population in 96-well V-bottom plates (3:1:1). HA29 and HI12 were added to respective wells at 4, 8, or 12 pM. Control wells contained the HLA-A2-scDb (pan A2, clone BB7.2) or isotype control H2-scDb (39) at 0.25 nM. Precision count beads (BioLegend) were added to each well for quantification of absolute cell numbers. Cells were pelleted and after incubation at 37 °C for 18 h, cocultures were stained with Fixable Viability Dye eFluor 780 (ThermoFisher). Samples were acquired on the Intellicyt and analyzed by FlowJo (Treestar).

The count of viable ALT-pulsed (CFSE^hi^) or ILK-pulsed (CTV^hi^) cells remaining after coculture was obtained by normalizing to the number of cells to the number of Precision Count Beads identified in the relevant well. For example, if 20,000 beads were plated and 10,000 beads were recovered upon flow cytometry acquisition, then the absolute count of cells in the well would be twice the amount in the flow gate. The percent reduction or cell death of a specific target was obtained by the following formula (shown for ALT-pulsed cells): (CFSE^hi^ cells without scDb − CFSE^hi^ cells with HA29-scDb)/(CFSE^hi^ cells without scDb × 100).

### In Vitro Infection Suppression Assay.

On day −10, PBMCs from healthy donors were stimulated with anti-human CD3 antibody (clone OKT3, BioLegend) for 3 d, then maintained in RPMI 1640 with 10% FBS, 1% penicillin/streptomycin, 250 U IL-2, and 5 ng/mL IL-7. On day −3, the CD8^+^ T cells were isolated from the stimulated PBMCs (StemCell). CD4^+^ T cells were also isolated from unstimulated, autologous PBMCs on day −3 (StemCell) and activated with CD3/CD28 Dynabeads (ThermoFisher). On day 0, activated CD4^+^ T cells were spinoculated with single-round ΔEnv-NL4.3-EGFP with ×4 Env at 400 ng p24/100,000 cells at 800 × *g* for 2 h at 37 °C. Infected and uninfected (pseudospinoculated) cells were rested at 37 °C for 2.5 h after spinoculation. scDbs were plated in 96-well U-bottom plates and preincubated with CD8^+^ T cells for 1 h in conditioned T cell media (STCM) prior to coculturing with CD4^+^ T cells at a 3:1 E:T ratio for 3 d at 37 °C. On day 3, culture supernatant was assayed for MIP1β using the Human CCL4/MIP-1B Quantikine ELISA (R&D Systems). Precision Count Beads (BioLegend) were added to sample wells. Cells were stained with Fixable Viability Dye eFluor 780 (ThermoFisher), Brilliant violet 605 anti-human CD8 Antibody (BioLegend), and Brilliant violet 421 anti-human CD3 Antibody (BioLegend) for 15 min at 4 °C. After washing cells, samples were acquired on an Intellicyt flow cytometer and analyzed with FlowJo software.

A similar procedure was used for suppression assays involving the 33A10 patient isolate. Activated CD4^+^ T cells from an HLA-A2^+^ healthy donor were spinoculated with 33A10 as above. Infected and uninfected (pseudospinoculated) cells were rested, washed with STCM, and then cocultured with autologous CD8^+^ T cells (prepared as above) at a 3:1 E:T ratio. The HA29-, HI12-, and H2-scDbs were added to the cocultures at 0.25 mM. Plates were spun at 800 RPM for 1 min and incubated for 3 d at 37 °C. Thirty microliters of supernatant were collected on days 1, 3, and 5 for measurement by p24 ELISA (PerkinElmer).

### Statistical Analysis.

Statistical analyses were carried out using the tests indicated in the figure legends. *P* < 0.05 is considered statistically significant. Analysis was performed using Prism v9 (GraphPad).

## Supplementary Material

Supplementary File

## Data Availability

All study data are included in the main text and *SI Appendix*.

## References

[1] T. W. Chun , In vivo fate of HIV-1-infected T cells: Quantitative analysis of the transition to stable latency. Nat. Med. 1, 1284–1290 (1995). 7489410 10.1038/nm1295-1284

[2] T. W. Chun , Quantification of latent tissue reservoirs and total body viral load in HIV-1 infection. Nature 387, 183–188 (1997). 9144289 10.1038/387183a0

[3] T. W. Chun, K. Chadwick, J. Margolick, R. F. Siliciano, Differential susceptibility of naive and memory CD4+ T cells to the cytopathic effects of infection with human immunodeficiency virus type 1 strain LAI. J. Virol. 71, 4436–4444 (1997). 9151834 10.1128/jvi.71.6.4436-4444.1997 PMC191662

[4] D. Finzi , Identification of a reservoir for HIV-1 in patients on highly active antiretroviral therapy. Science 278, 1295–1300 (1997). 9360927 10.1126/science.278.5341.1295

[5] J. K. Wong , Recovery of replication-competent HIV despite prolonged suppression of plasma viremia. Science 278, 1291–1295 (1997). 9360926 10.1126/science.278.5341.1291

[6] R. T. Davey Jr , HIV-1 and T cell dynamics after interruption of highly active antiretroviral therapy (HAART) in patients with a history of sustained viral suppression. Proc. Natl. Acad. Sci. U.S.A. 96, 15109–15114 (1999). 10611346 10.1073/pnas.96.26.15109 PMC24781

[7] T. W. Chun, R. T. Davey Jr., D. Engel, H. C. Lane, A. S. Fauci, Re-emergence of HIV after stopping therapy. Nature 401, 874–875 (1999). 10553903 10.1038/44755

[8] D. Finzi , Latent infection of CD4+ T cells provides a mechanism for lifelong persistence of HIV-1, even in patients on effective combination therapy. Nat. Med. 5, 512–517 (1999). 10229227 10.1038/8394

[9] J. D. Siliciano , Long-term follow-up studies confirm the stability of the latent reservoir for HIV-1 in resting CD4+ T cells. Nat. Med. 9, 727–728 (2003). 12754504 10.1038/nm880

[10] M. C. Strain , Heterogeneous clearance rates of long-lived lymphocytes infected with HIV: Intrinsic stability predicts lifelong persistence. Proc. Natl. Acad. Sci. U.S.A. 100, 4819–4824 (2003). 12684537 10.1073/pnas.0736332100 PMC153639

[11] A. M. Crooks , Precise quantitation of the latent HIV-1 reservoir: Implications for eradication strategies. J. Infect. Dis. 212, 1361–1365 (2015). 25877550 10.1093/infdis/jiv218 PMC4601910

[12] N. M. Archin, J. M. Sung, C. Garrido, N. Soriano-Sarabia, D. M. Margolis, Eradicating HIV-1 infection: Seeking to clear a persistent pathogen. Nat. Rev. Microbiol. 12, 750–764 (2014). 25402363 10.1038/nrmicro3352 PMC4383747

[13] A. R. M. Townsend , The epitopes of influenza nucleoprotein recognized by cytotoxic T lymphocytes can be defined with short synthetic peptides. Cell 44, 959–968 (1986). 2420472 10.1016/0092-8674(86)90019-x

[14] N. M. Archin , Administration of vorinostat disrupts HIV-1 latency in patients on antiretroviral therapy. Nature 487, 482–485 (2012). 22837004 10.1038/nature11286 PMC3704185

[15] J. H. Elliott , Activation of HIV transcription with short-course vorinostat in HIV-infected patients on suppressive antiretroviral therapy. PLoS Pathog. 10, e1004473 (2014). 25393648 10.1371/journal.ppat.1004473 PMC4231123

[16] T. A. Rasmussen , Panobinostat, a histone deacetylase inhibitor, for latent-virus reactivation in HIV-infected patients on suppressive antiretroviral therapy: A phase 1/2, single group, clinical trial. Lancet HIV 1, e13–e21 (2014). 26423811 10.1016/S2352-3018(14)70014-1

[17] O. S. Søgaard , The depsipeptide romidepsin reverses HIV-1 latency in vivo. PLoS Pathog. 11, e1005142 (2015). 26379282 10.1371/journal.ppat.1005142 PMC4575032

[18] S. A. Migueles , Defective human immunodeficiency virus-specific CD8+ T-cell polyfunctionality, proliferation, and cytotoxicity are not restored by antiretroviral therapy. J. Virol. 83, 11876–11889 (2009). 19726501 10.1128/JVI.01153-09 PMC2772718

[19] M. R. Betts , HIV nonprogressors preferentially maintain highly functional HIV-specific CD8+ T cells. Blood 107, 4781–4789 (2006). 16467198 10.1182/blood-2005-12-4818 PMC1895811

[20] L. Shan , Stimulation of HIV-1-specific cytolytic T lymphocytes facilitates elimination of latent viral reservoir after virus reactivation. Immunity 36, 491–501 (2012). 22406268 10.1016/j.immuni.2012.01.014 PMC3501645

[21] K. Deng , Broad CTL response is required to clear latent HIV-1 due to dominance of escape mutations. Nature 517, 381–385 (2015). 25561180 10.1038/nature14053 PMC4406054

[22] R. B. Jones , Histone deacetylase inhibitors impair the elimination of HIV-infected cells by cytotoxic T-lymphocytes. PLoS Pathog. 10, e1004287 (2014). 25122219 10.1371/journal.ppat.1004287 PMC4133386

[23] R. B. Jones , A subset of latency-reversing agents expose HIV-infected resting CD4+ T-cells to recognition by cytotoxic T-lymphocytes. PLoS Pathog. 12, e1005545 (2016). 27082643 10.1371/journal.ppat.1005545 PMC4833318

[24] V. E. Walker-Sperling, C. W. Pohlmeyer, P. M. Tarwater, J. N. Blankson, The effect of latency reversal agents on primary CD8+ T cells: Implications for shock and kill strategies for human immunodeficiency virus eradication. EBioMedicine 8, 217–229 (2016). 27428432 10.1016/j.ebiom.2016.04.019 PMC4919475

[25] A. K. Kwaa , The effect of Ingenol-B on the suppressive capacity of elite suppressor HIV-specific CD8+ T cells. PLoS One 12, e0174516 (2017). 28467486 10.1371/journal.pone.0174516 PMC5414940

[26] S. L. Maude , Chimeric antigen receptor T cells for sustained remissions in leukemia. N. Engl. J. Med. 371, 1507–1517 (2014). 25317870 10.1056/NEJMoa1407222 PMC4267531

[27] M. Sadelain, I. Rivière, S. Riddell, Therapeutic T cell engineering. Nature 545, 423–431 (2017). 28541315 10.1038/nature22395 PMC5632949

[28] C. H. June, M. Sadelain, Chimeric antigen receptor therapy. N. Engl. J. Med. 379, 64–73 (2018). 29972754 10.1056/NEJMra1706169 PMC7433347

[29] M. Subklewe, BiTEs better than CAR T cells. Blood Adv. 5, 607–612 (2021). 33496755 10.1182/bloodadvances.2020001792 PMC7839370

[30] H. Kantarjian , Blinatumomab versus chemotherapy for advanced acute lymphoblastic leukemia. N. Engl. J. Med. 376, 836–847 (2017). 28249141 10.1056/NEJMoa1609783 PMC5881572

[31] T. K. Bera, P. E. Kennedy, E. A. Berger, C. F. Barbas 3rd, I. Pastan, Specific killing of HIV-infected lymphocytes by a recombinant immunotoxin directed against the HIV-1 envelope glycoprotein. Mol. Med. 4, 384–391 (1998). 10780881 PMC2230270

[32] G. Ferrari , Envelope-specific antibodies and antibody-derived molecules for treating and curing HIV infection. Nat. Rev. Drug Discov. 15, 823–834 (2016). 27725635 10.1038/nrd.2016.173 PMC5549020

[33] D. D. Sloan , Targeting HIV reservoir in infected CD4 T cells by Dual-Affinity Re-targeting Molecules (DARTs) that bind HIV envelope and recruit cytotoxic T cells. PLoS Pathog. 11, e1005233 (2015). 26539983 10.1371/journal.ppat.1005233 PMC4634948

[34] J. A. M. Sung , Dual-Affinity Re-Targeting proteins direct T cell-mediated cytolysis of latently HIV-infected cells. J. Clin. Invest. 125, 4077–4090 (2015). 26413868 10.1172/JCI82314 PMC4639974

[35] C. C. LaBranche , A single amino acid change in the cytoplasmic domain of the simian immunodeficiency virus transmembrane molecule increases envelope glycoprotein expression on infected cells. J. Virol. 69, 5217–5227 (1995). 7636963 10.1128/jvi.69.9.5217-5227.1995 PMC189351

[36] R. Byland, P. J. Vance, J. A. Hoxie, M. Marsh, A conserved dileucine motif mediates clathrin and AP-2-dependent endocytosis of the HIV-1 envelope protein. Mol. Biol. Cell 18, 414–425 (2007). 17108326 10.1091/mbc.E06-06-0535 PMC1783771

[37] M. J. Hogan , Increased surface expression of HIV-1 envelope is associated with improved antibody response in vaccinia prime/protein boost immunization. Virology 514, 106–117 (2018). 29175625 10.1016/j.virol.2017.10.013 PMC5770335

[38] J. Douglass , Bispecific antibodies targeting mutant *RAS* neoantigens. Sci. Immunol. 6, eabd5515 (2021). 33649101 10.1126/sciimmunol.abd5515 PMC8141259

[39] E. H. C. Hsiue , Targeting a neoantigen derived from a common *TP53* mutation. Science 371, eabc8697 (2021). 33649166 10.1126/science.abc8697 PMC8208645

[40] P. D. Holler, L. K. Chlewicki, D. M. Kranz, TCRs with high affinity for foreign pMHC show self-reactivity. Nat. Immunol. 4, 55–62 (2003). 12469116 10.1038/ni863

[41] F. F. González-Galarza , Allele frequency net 2015 update: New features for HLA epitopes, KIR and disease and HLA adverse drug reaction associations. Nucleic Acids Res. 43, D784–D788 (2015). 25414323 10.1093/nar/gku1166 PMC4383964

[42] M. Andreatta, M. Nielsen, Gapped sequence alignment using artificial neural networks: Application to the MHC class I system. Bioinformatics 32, 511–517 (2016). 26515819 10.1093/bioinformatics/btv639 PMC6402319

[43] D. B. Keskin , Physical detection of influenza A epitopes identifies a stealth subset on human lung epithelium evading natural CD8 immunity. Proc. Natl. Acad. Sci. U.S.A. 112, 2151–2156 (2015). 25646416 10.1073/pnas.1423482112 PMC4343122

[44] B. Reinhold, D. B. Keskin, E. L. Reinherz, Molecular detection of targeted major histocompatibility complex I-bound peptides using a probabilistic measure and nanospray MS3 on a hybrid quadrupole-linear ion trap. Anal. Chem. 82, 9090–9099 (2010). 20932029 10.1021/ac102387t PMC2966287

[45] B. Reynisson, B. Alvarez, S. Paul, B. Peters, M. Nielsen, NetMHCpan-4.1 and NetMHCIIpan-4.0: Improved predictions of MHC antigen presentation by concurrent motif deconvolution and integration of MS MHC eluted ligand data. Nucleic Acids Res. 48, W449–W454 (2020). 32406916 10.1093/nar/gkaa379 PMC7319546

[46] H. Zhang , Novel single-cell-level phenotypic assay for residual drug susceptibility and reduced replication capacity of drug-resistant human immunodeficiency virus type 1. J. Virol. 78, 1718–1729 (2004). 14747537 10.1128/JVI.78.4.1718-1729.2004 PMC369469

[47] Y. Sykulev, M. Joo, I. Vturina, T. J. Tsomides, H. N. Eisen, Evidence that a single peptide-MHC complex on a target cell can elicit a cytolytic T cell response. Immunity 4, 565–571 (1996). 8673703 10.1016/s1074-7613(00)80483-5

[48] M. A. Purbhoo, D. J. Irvine, J. B. Huppa, M. M. Davis, T cell killing does not require the formation of a stable mature immunological synapse. Nat. Immunol. 5, 524–530 (2004). 15048111 10.1038/ni1058

[49] R. Dahan, Y. Reiter, T-cell-receptor-like antibodies—Generation, function and applications. Expert Rev. Mol. Med. 14, e6 (2012). 22361332 10.1017/erm.2012.2

[50] A. D. Skora , Generation of MANAbodies specific to HLA-restricted epitopes encoded by somatically mutated genes. Proc. Natl. Acad. Sci. U.S.A. 112, 9967–9972 (2015). 26216968 10.1073/pnas.1511996112 PMC4538619

[51] M. S. Miller , An engineered antibody fragment targeting mutant β-catenin via major histocompatibility complex I neoantigen presentation. J. Biol. Chem. 294, 19322–19334 (2019). 31690625 10.1074/jbc.RA119.010251 PMC6916501

[52] R. A. Seder, P. A. Darrah, M. Roederer, T-cell quality in memory and protection: Implications for vaccine design. Nat. Rev. Immunol. 8, 247–258 (2008). 18323851 10.1038/nri2274

[53] M. E. May , Combined effects of HLA-B*57/5801 elite suppressor CD8+ T cells and NK cells on HIV-1 replication. Front. Cell. Infect. Microbiol. 10, 113 (2020). 32266164 10.3389/fcimb.2020.00113 PMC7098910

[54] G. B. Cohen , The selective downregulation of class I major histocompatibility complex proteins by HIV-1 protects HIV-infected cells from NK cells. Immunity 10, 661–671 (1999). 10403641 10.1016/s1074-7613(00)80065-5

[55] T. Kerkau , The human immunodeficiency virus type 1 (HIV-1) Vpu protein interferes with an early step in the biosynthesis of major histocompatibility complex (MHC) class I molecules. J. Exp. Med. 185, 1295–1305 (1997). 9104816 10.1084/jem.185.7.1295 PMC2196253

[56] Y. C. Ho , Replication-competent noninduced proviruses in the latent reservoir increase barrier to HIV-1 cure. Cell 155, 540–551 (2013). 24243014 10.1016/j.cell.2013.09.020 PMC3896327

[57] O. Lambotte ; SEROCO-HEMOCO Study Group, HIV controllers: A homogeneous group of HIV-1-infected patients with spontaneous control of viral replication. Clin. Infect. Dis. 41, 1053–1056 (2005). 16142675 10.1086/433188

[58] S. Grabar , Prevalence and comparative characteristics of long-term nonprogressors and HIV controller patients in the French Hospital Database on HIV. AIDS 23, 1163–1169 (2009). 19444075 10.1097/QAD.0b013e32832b44c8

[59] K. A. O’Connell, J. R. Bailey, J. N. Blankson, Elucidating the elite: Mechanisms of control in HIV-1 infection. Trends Pharmacol. Sci. 30, 631–637 (2009). 19837464 10.1016/j.tips.2009.09.005

[60] A. Sáez-Cirión ; Agence Nationale de Recherches sur le Sida EP36 HIV Controllers Study Group, HIV controllers exhibit potent CD8 T cell capacity to suppress HIV infection ex vivo and peculiar cytotoxic T lymphocyte activation phenotype. Proc. Natl. Acad. Sci. U.S.A. 104, 6776–6781 (2007). 17428922 10.1073/pnas.0611244104 PMC1851664

[61] G. D. Gaiha , Structural topology defines protective CD8^+^ T cell epitopes in the HIV proteome. Science 364, 480–484 (2019). 31048489 10.1126/science.aav5095 PMC6855781

[62] V. Appay , HIV-specific CD8(+) T cells produce antiviral cytokines but are impaired in cytolytic function. J. Exp. Med. 192, 63–75 (2000). 10880527 10.1084/jem.192.1.63 PMC1887711

[63] S. A. Migueles , Lytic granule loading of CD8+ T cells is required for HIV-infected cell elimination associated with immune control. Immunity 29, 1009–1021 (2008). 19062316 10.1016/j.immuni.2008.10.010 PMC2622434

[64] G. Bossi , Examining the presentation of tumor-associated antigens on peptide-pulsed T2 cells. OncoImmunology 2, e26840 (2013). 24482751 10.4161/onci.26840 PMC3894244

[65] T. J. Tsomides , Naturally processed viral peptides recognized by cytotoxic T lymphocytes on cells chronically infected by human immunodeficiency virus type 1. J. Exp. Med. 180, 1283–1293 (1994). 7523570 10.1084/jem.180.4.1283 PMC2191672

[66] F. Kirchhoff, Immune evasion and counteraction of restriction factors by HIV-1 and other primate lentiviruses. Cell Host Microbe 8, 55–67 (2010). 20638642 10.1016/j.chom.2010.06.004

[67] Y. Dong , Cryo-EM structures and dynamics of substrate-engaged human 26S proteasome. Nature 565, 49–55 (2019). 30479383 10.1038/s41586-018-0736-4 PMC6370054

[68] J. W. Yewdell, E. Reits, J. Neefjes, Making sense of mass destruction: Quantitating MHC class I antigen presentation. Nat. Rev. Immunol. 3, 952–961 (2003). 14647477 10.1038/nri1250

[69] P. Borrow , Antiviral pressure exerted by HIV-1-specific cytotoxic T lymphocytes (CTLs) during primary infection demonstrated by rapid selection of CTL escape virus. Nat. Med. 3, 205–211 (1997). 9018240 10.1038/nm0297-205

[70] N. Goonetilleke ; CHAVI Clinical Core B, The first T cell response to transmitted/founder virus contributes to the control of acute viremia in HIV-1 infection. J. Exp. Med. 206, 1253–1272 (2009). 19487423 10.1084/jem.20090365 PMC2715063

[71] B. D. Walker , HIV-specific cytotoxic T lymphocytes in seropositive individuals. Nature 328, 345–348 (1987). 3496541 10.1038/328345a0

[72] B. D. Walker , HIV-1 reverse transcriptase is a target for cytotoxic T lymphocytes in infected individuals. Science 240, 64–66 (1988). 2451288 10.1126/science.2451288

[73] M. A. Checkley, B. G. Luttge, E. O. Freed, HIV-1 envelope glycoprotein biosynthesis, trafficking, and incorporation. J. Mol. Biol. 410, 582–608 (2011). 21762802 10.1016/j.jmb.2011.04.042 PMC3139147

[74] W. Fischer ; Network for Genomic Surveillance in South Africa (NGS-SA), HIV-1 and SARS-CoV-2: Patterns in the evolution of two pandemic pathogens. Cell Host Microbe 29, 1093–1110 (2021). 34242582 10.1016/j.chom.2021.05.012 PMC8173590

[75] D. R. Burton, L. Hangartner, Broadly neutralizing antibodies to HIV and their role in vaccine design. Annu. Rev. Immunol. 34, 635–659 (2016). 27168247 10.1146/annurev-immunol-041015-055515 PMC6034635

[76] J. F. Scheid , HIV-1 antibody 3BNC117 suppresses viral rebound in humans during treatment interruption. Nature 535, 556–560 (2016). 27338952 10.1038/nature18929 PMC5034582

[77] S. G. Hansen , Effector memory T cell responses are associated with protection of rhesus monkeys from mucosal simian immunodeficiency virus challenge. Nat. Med. 15, 293–299 (2009). 19219024 10.1038/nm.1935 PMC2720091

[78] S. G. Hansen , Immune clearance of highly pathogenic SIV infection. Nature 502, 100–104 (2013). 24025770 10.1038/nature12519 PMC3849456

[79] S. G. Hansen , Broadly targeted CD8^+^ T cell responses restricted by major histocompatibility complex E. Science 351, 714–720 (2016). 26797147 10.1126/science.aac9475 PMC4769032

[80] C. K. Bullen, G. M. Laird, C. M. Durand, J. D. Siliciano, R. F. Siliciano, New ex vivo approaches distinguish effective and ineffective single agents for reversing HIV-1 latency in vivo. Nat. Med. 20, 425–429 (2014). 24658076 10.1038/nm.3489 PMC3981911

[81] D. Banik , Single molecule force spectroscopy reveals distinctions in key biophysical parameters of αβ T-cell receptors compared with chimeric antigen receptors directed at the same ligand. J. Phys. Chem. Lett. 12, 7566–7573 (2021). 34347491 10.1021/acs.jpclett.1c02240 PMC9082930

[82] D. K. Das , Force-dependent transition in the T-cell receptor β-subunit allosterically regulates peptide discrimination and pMHC bond lifetime. Proc. Natl. Acad. Sci. U.S.A. 112, 1517–1522 (2015). 25605925 10.1073/pnas.1424829112 PMC4321250

[83] Y. Feng , Mechanosensing drives acuity of *αβ* T-cell recognition. Proc. Natl. Acad. Sci. U.S.A. 114, E8204–E8213 (2017). 28811364 10.1073/pnas.1703559114 PMC5625899

[84] Y. Feng, E. L. Reinherz, M. J. Lang, αβ T cell receptor mechanosensing forces out serial engagement. Trends Immunol. 39, 596–609 (2018). 30060805 10.1016/j.it.2018.05.005 PMC6154790

[85] K. L. Hui, L. Balagopalan, L. E. Samelson, A. Upadhyaya, Cytoskeletal forces during signaling activation in Jurkat T-cells. Mol. Biol. Cell 26, 685–695 (2015). 25518938 10.1091/mbc.E14-03-0830 PMC4325839

[86] J. Husson, K. Chemin, A. Bohineust, C. Hivroz, N. Henry, Force generation upon T cell receptor engagement. PLoS One 6, e19680 (2011). 21572959 10.1371/journal.pone.0019680 PMC3091878

[87] S. T. Kim , The alphabeta T cell receptor is an anisotropic mechanosensor. J. Biol. Chem. 284, 31028–31037 (2009). 19755427 10.1074/jbc.M109.052712 PMC2781503

[88] Y.-C. Li , Cutting edge: Mechanical forces acting on T cells immobilized via the TCR complex can trigger TCR signaling. J. Immunol. 184, 5959–5963 (2010). 20435924 10.4049/jimmunol.0900775

[89] B. Liu, W. Chen, B. D. Evavold, C. Zhu, Accumulation of dynamic catch bonds between TCR and agonist peptide-MHC triggers T cell signaling. Cell 157, 357–368 (2014). 24725404 10.1016/j.cell.2014.02.053 PMC4123688

[90] L. V. Sibener , Isolation of a structural mechanism for uncoupling T cell receptor signaling from peptide-MHC binding. Cell 174, 672–687.e27 (2018). 30053426 10.1016/j.cell.2018.06.017 PMC6140336

[91] A. Rodari, G. Darcis, C. M. Van Lint, The current status of latency reversing agents for HIV-1 remission. Annu. Rev. Virol. 8, 491–514 (2021). 34586875 10.1146/annurev-virology-091919-103029

[92] K. L. Clayton , HIV-infected macrophages resist efficient NK cell-mediated killing while preserving inflammatory cytokine responses. Cell Host Microbe 29, 435–447.e9 (2021). 33571449 10.1016/j.chom.2021.01.006 PMC8486985

[93] Y. Fukazawa , B cell follicle sanctuary permits persistent productive simian immunodeficiency virus infection in elite controllers. Nat. Med. 21, 132–139 (2015). 25599132 10.1038/nm.3781 PMC4320022

[94] S. H. Huang , Latent HIV reservoirs exhibit inherent resistance to elimination by CD8+ T cells. J. Clin. Invest. 128, 876–889 (2018). 29355843 10.1172/JCI97555 PMC5785246

[95] C. L. Day , PD-1 expression on HIV-specific T cells is associated with T-cell exhaustion and disease progression. Nature 443, 350–354 (2006). 16921384 10.1038/nature05115

[96] E. N. Borducchi , Ad26/MVA therapeutic vaccination with TLR7 stimulation in SIV-infected rhesus monkeys. Nature 540, 284–287 (2016). 27841870 10.1038/nature20583 PMC5145754

[97] E. N. Borducchi , Antibody and TLR7 agonist delay viral rebound in SHIV-infected monkeys. Nature 563, 360–364 (2018). 30283138 10.1038/s41586-018-0600-6 PMC6237629

