# Supplementary material for: TCR-mimic bispecific antibodies to target the HIV-1 reservoir
Source: Proc Natl Acad Sci U S A. 2022 Apr 8;119(15):e2123406119. doi: 10.1073/pnas.2123406119 (PMC9169739; doi:10.1073/pnas.2123406119)
Supplement: Supplementary File [file pnas.2123406119.sapp.pdf]

PNAS
www.pnas.org

Supplementary Information for

TCR-mimic bispecific antibodies to target the HIV-1 reservoir

Srona Sengupta[1,2 †], Nathan Board[1, ‡], Fengting Wu[1, ‡], Milica Moskovljevic[1, ‡], Jacqueline Douglass[3,4,‡], Josephine Zhang[1], Bruce R. Reinhold[5-7], Jonathan Duke-Cohan[5-7], Jeanna Yu[1], Madison C. Reed[1], Yasmine Tabdili[1], Aitana Azurmendi[8], Emily J. Fray[1], Hao Zhang[9], Emily Han-Chung Hsiue[3,4], Katharine Jenike[1], Ya-chi Ho[10], Sandra B. Gabelli[8], Kenneth W. Kinzler[3,4,11], Bert Vogelstein[3,4,11,12], Shibin Zhou[3,4,11], Janet D. Siliciano[1], Scheherazade Sadegh-Nasseri[2], Ellis L. Reinherz[5-7] , and Robert F. Siliciano[1,12,*].

‡ These authors contributed equally.

*Corresponding author.

Contributed by Robert F. Siliciano

To whom correspondence should be addressed**:**

Email: rsiliciano@jhmi.edu (R.F.S.)

This PDF file includes:

Methods for phage screening
Supplementary Figures 1-9
Tables S1 to S3
SI References

Phage panning and characterization

Phage panning was performed as previously described (1). Briefly, panning consisted of four rounds of negative and positive selection. Negative selection was performed against naked streptavidin beads, free streptavidin, and irrelevant, and biotinylated A2-pMHC pre-conjugated to streptavidin beads. Negative selection was also performed against A2-bearing cells (T2s, RPMI-6666s). Positive selection was performed for each round of panning with decreasing amounts of the relevant biotinylated monomer conjugated to streptavidin beads. After each round of panning, phage were eluted with glycine (pH 2), neutralized with Tris-HCL (pH 9), amplified in SS320 cells (Lucigen), and concentrated in PEG-NaCl. Phage eluted from the 4th round of panning were diluted such that infection of bacteria produced single colonies. Individual bacterial colonies were inoculated in deep 96-well plates to produce monoclonal phage. Monoclonal phage supernatants were tested for their ability to bind to the target versus irrelevant pMHC using ELISA and flow cytometry. Phage supernatants were added to streptavidin ELISA plates pre-coated with biotinylated A2-monomers of the relevant or irrelevant pMHC, and phage binding was assessed using a rabbit anti-M13 antibody (Pierce) and a secondary conjugated to HRP. To assess phage binding to pMHC on the surface of cells, T2 cells were pulsed with 50 ug/ml peptide and 10 ug/ml B2M for 4 hrs to overnight at 37°C in RPMI 1640 + Glutamax with 1% Pen/Strep. Pulsed cells were washed once with PBS, incubated with monoclonal phage, and then stained with rabbit anti-M13 (Pierce) followed by a PE-conjugated donkey anti-rabbit secondary (Biolegend). Phage-stained cells were acquired on the Intellicyt flow cytometer and analyzed with FlowJo.

scDb Expression and Purification

Monoclonal phages that stained specifically for a particular peptide-pulsed target were sequenced to identify the expressed Fab. Gene blocks expressing this Fab fragment, the UCHT1 Fab fragment against CD3, and C'terminal His tag were cloned via Gibson assembly into the pcDNA3.4 vector backbone. scDb constructs were sequenced, amplified, and expressed via transfection into 293T cells. scDbs were purified using nickel columns (Capturem His-tag miniprep columns) using 400 mM imidazole and were desalted to remove imidazole using Zeba Spin columns. scDbs were run on SDS-PAGE and quantified using iBright densitometric analysis in comparison to a BSA standard. Large-scale preparations of scDbs were produced by ThermoFisher.

S1

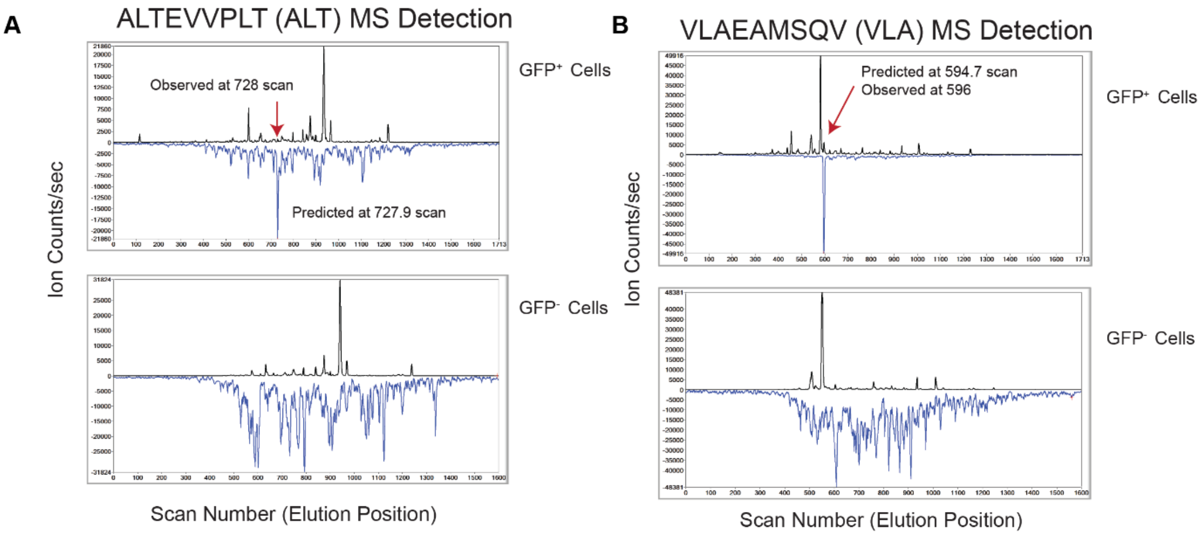

Fig. S1: Poisson LC-DIAMS detection of HLA-A*02:01-bound ALT and VLA peptides from 2.5x10^6 GFP$^+$ CD4$^+$T cells. A, B) Detection of ALTEVVPLT (ALT) and VLAEAMSQV (VLA) HIV-1 peptides in GFP$^+$ cells, indicated by the presence of coeluting peaks in the precursor XIC (top black trace) and the Poisson chromatogram (bottom, inverted blue trace). The elution position of the coincident peaks must also be consistent with the elution mapping as determined by an LC-DIAMS run of the synthetic set containing a set of mapping peptides shared in both the synthetic and sample runs (see Methods). Of the 64 HIV-1 HLA-A*02:01-binding peptides assayed, these two were the only ones detected in the +2-charge state. None of the 64 were detected in GFP$^-$ cells.

S2

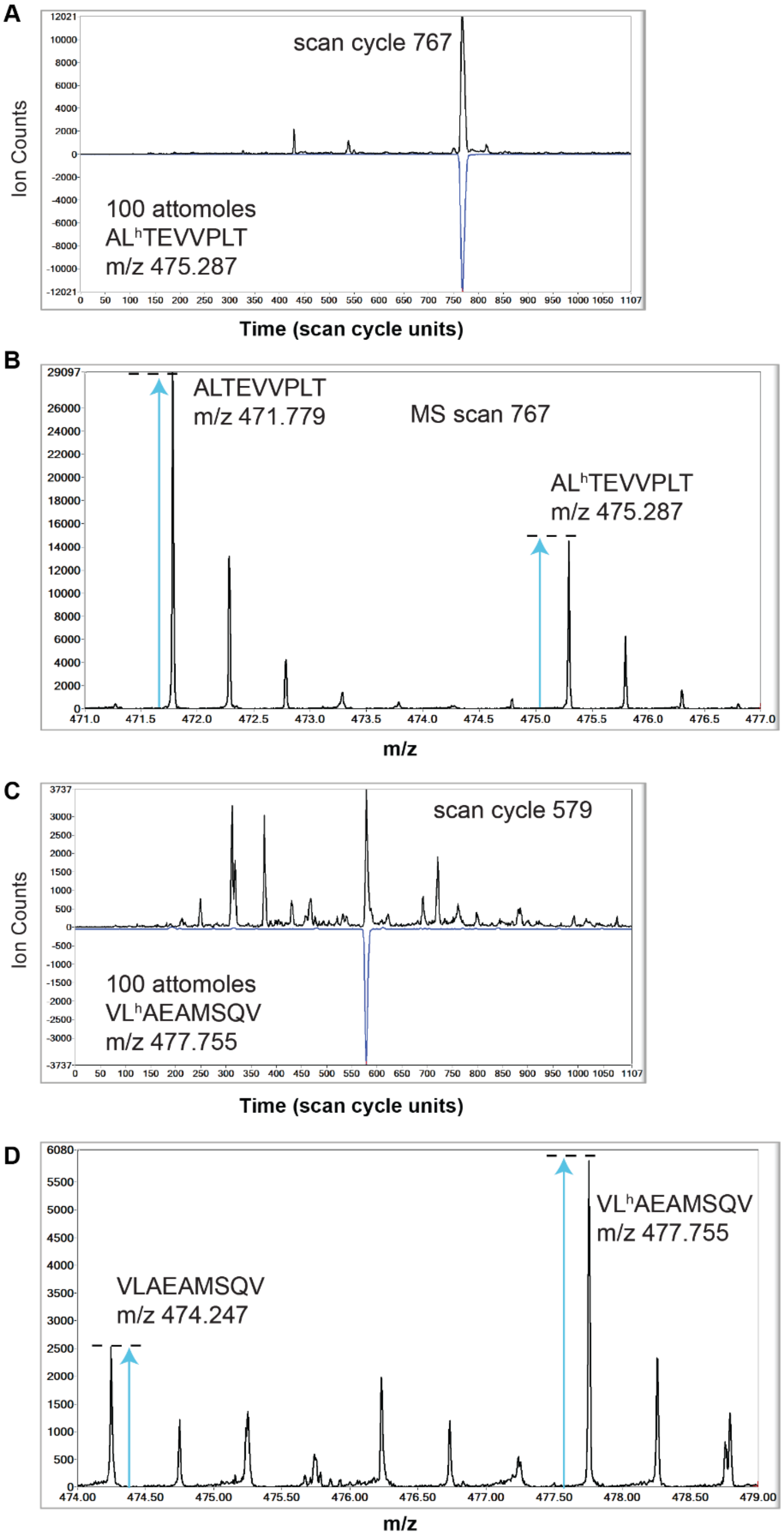
A
Ion Counts
scan cycle 767
100 attomoles
AL^hTEVVPLT
m/z 475.287
Time (scan cycle units)
B
ALTEVVPLT
m/z 471.779
MS scan 767
AL^hTEVVPLT
m/z 475.287
m/z
C
Ion Counts
scan cycle 579
100 attomoles
VL^hAEAMSQV
m/z 477.755
Time (scan cycle units)
D
VL^hAEAMSQV
m/z 477.755
VLAEAMSQV
m/z 474.247
m/z

Fig. S2: Quantitation of HLA-A*02:01-bound peptides ALT and VLA detected from 2.5e6 GFP^{+} CD4^{+}T cell by targeted LC-MS/MS. 100 attomoles of isotope-labeled heavy ALT and VLA peptides were added. **A**) Poisson detection plot shows heavy ALT, hence light ALT, eluting at scan 767. **B**) The mass spectrum at scan 767 shows precursor ion amplitudes for both light (30,000) and heavy (13,300) forms. 30000/13300 * 100 = 226 attomoles. Over 2.5 million cells this is a copy number of 54 per cell. **C, D**) As above with 2500/5850*100 = 43 attomoles or 10 copies/cell.

S3

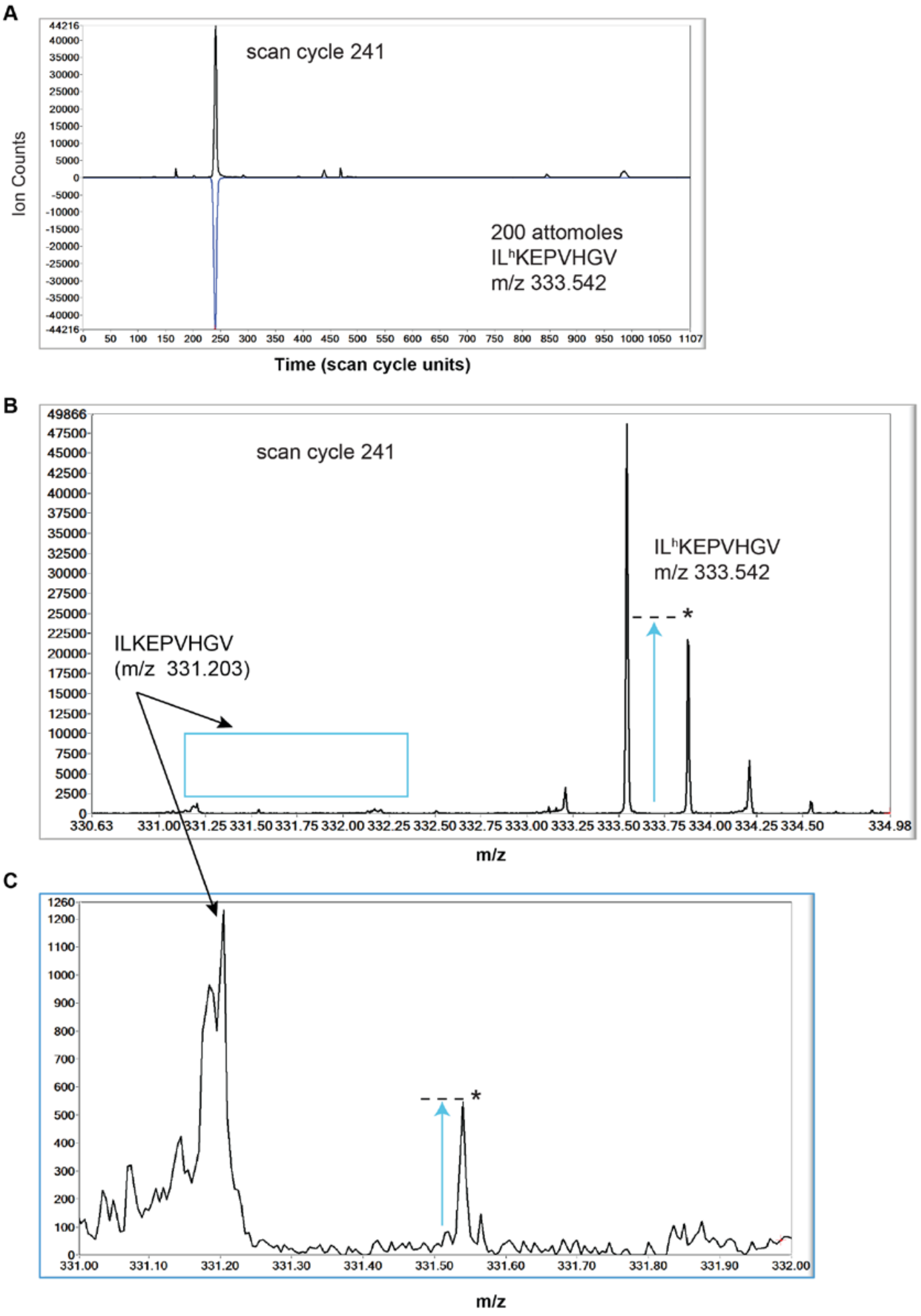

Fig. S3: Quantitation of HLA-A*02:01-bound ILK detected from 2.5e6 GFP^{+} CD4^{+}T cell by targeted LC-MS/MS. 200 attomoles of isotope-labeled heavy ILK were added. **A**) Poisson detection plot shows

heavy ILK, hence light ILK, eluting at scan 241. **B**) The mass spectrum at scan 241 shows the precursor ion amplitude of the heavy form but to see the light form light form requires an expanded scale (**C**). That this minor peak is light ILK is shown by its Poisson signature in Fig. 1. The base isotope peak at 331.203 is overlapping with background ions, hence the first isotope peaks (marked with asterisks) is used for quantitation. 570/22400 * 200 = 5 attomoles which is less than 1 copy/cell.

S4

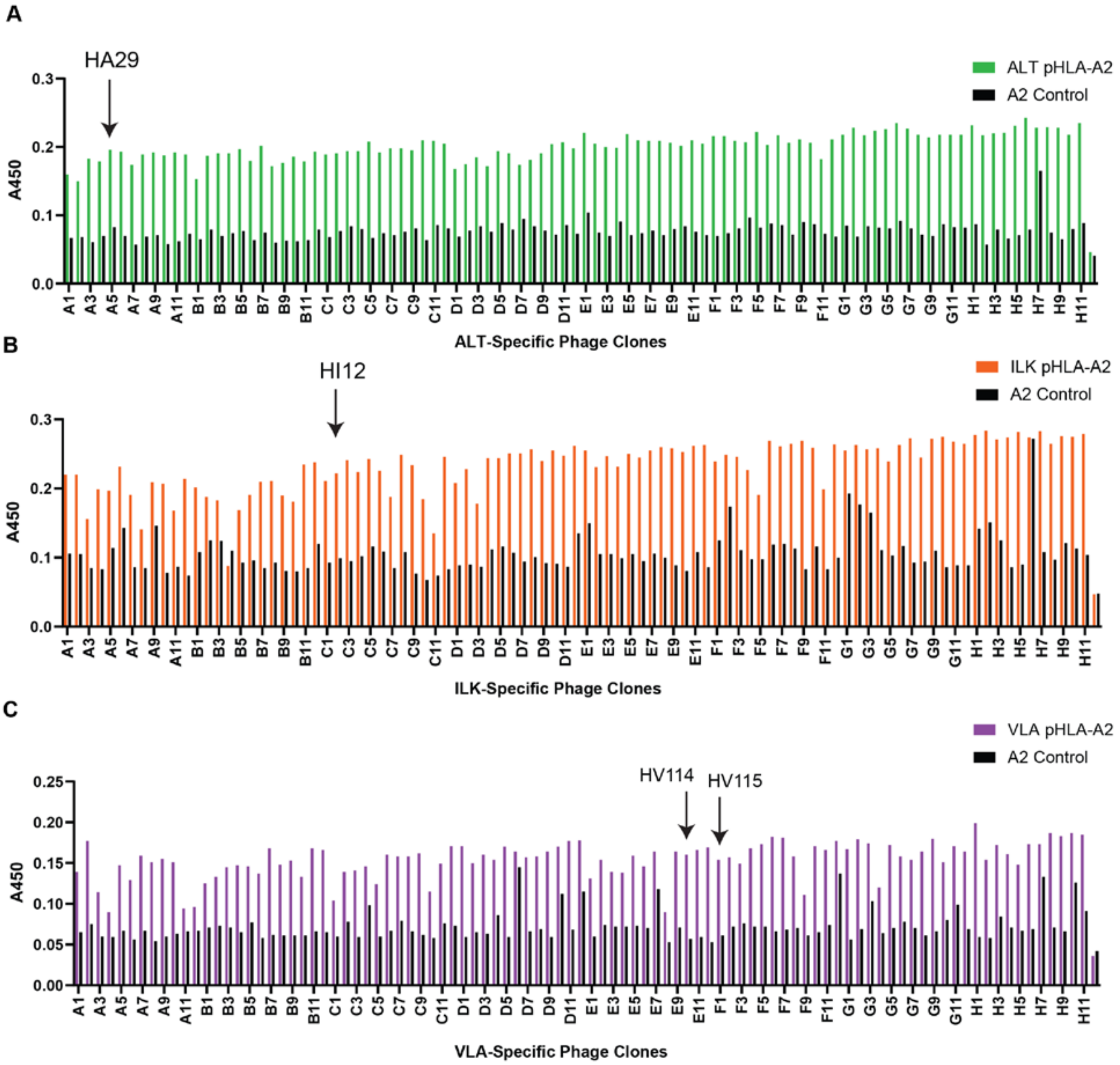

Fig. S4: Selection of scFv specific for ALT, ILK, and VLA pMHC. A) Monoclonal phage were amplified in bacteria in a 96-well plate format after 4 rounds of selection and then incubated in streptavidin ELISA plates coated with biotinylated ALT pHLA-A2, ILK pHLA-A2, VLA pHLA-A2, or an isotype pHLA-A2 control. Plates were washed and phage were detected by absorbance at 450 nm using rabbit anti-M13 and an HRP-conjugated mouse anti-rabbit secondary antibody. Arrows indicate phage whose scFv sequences were utilized to construct the HA29, HI12, HV114, and HV115 scDbs described in the manuscript. Well H12 was not inoculated with phage and therefore served as a no phage control.

S5

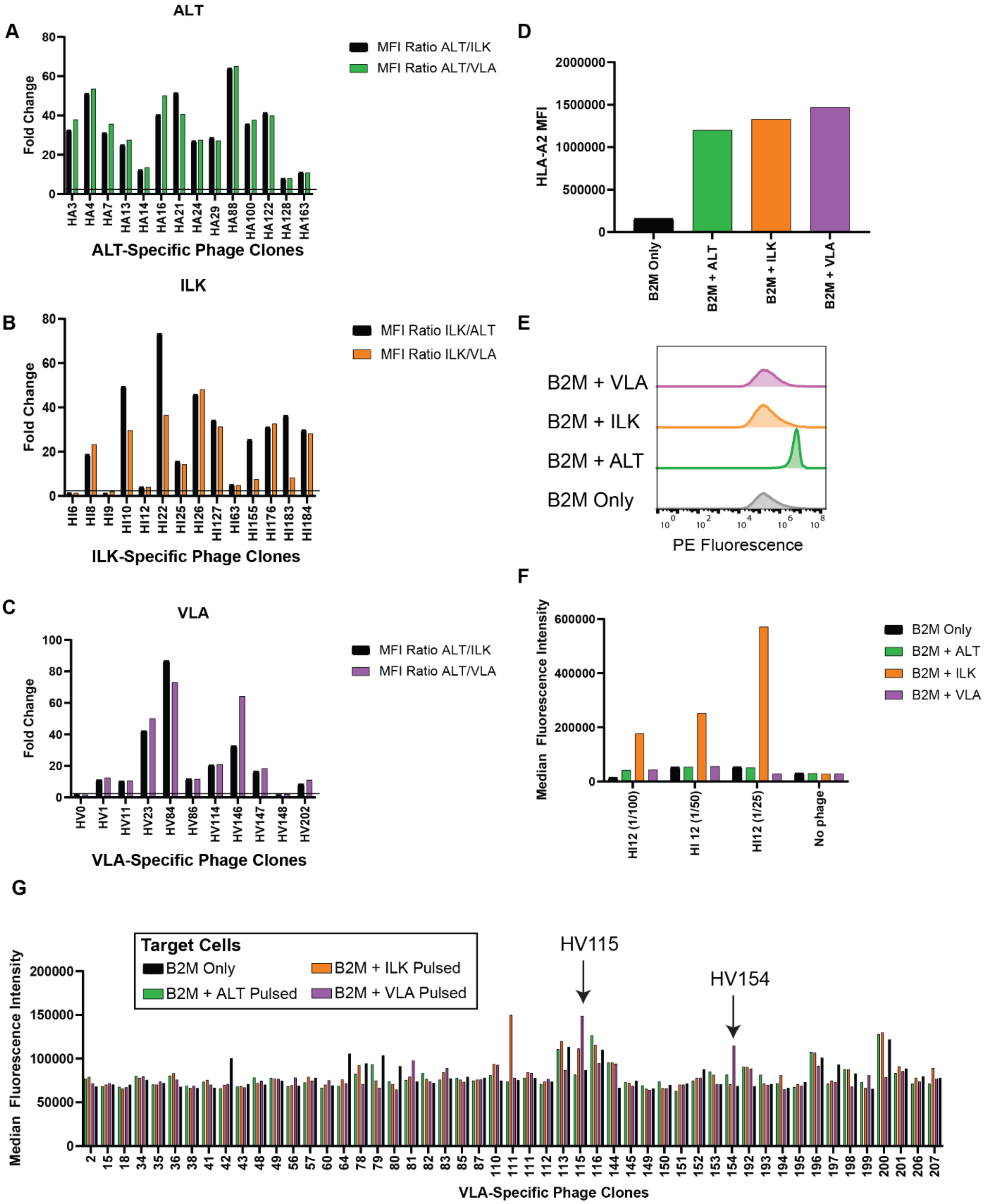

Fig. S5: Flow cytometry characterization of specific monoclonal phage. pMHC-specific monoclonal phage clones that bound >2 fold higher to the target vs irrelevant pMHC-A2 in monoclonal ELISAs were tested for binding on T2 (HLA-A*02:01^{+}) cells pulsed with 50 ug/ml of the indicated peptide. Phage binding was assessed by flow cytometry using a rabbit anti-M13 primary antibody and a donkey anti-Rabbit secondary antibody conjugated to phycoerythrin (PE). Phage binding was quantified by PE

median fluorescence intensity (MFI), and the ratio of monoclonal phage bound to cells pulsed with the cognate or irrelevant peptides plus beta-2 microglobulin (B2M) was used to rank pMHC-specific phage by specificity. **A-C**) The MFI ratio of HA, HI, or HV-specific phage binding to cognate (ALT, ILK, or VLA peptides, respectively) versus irrelevant peptide-pulsed cells is plotted. **D**) Peptide stabilization of cell-surface HLA-A2 on T2 cells is shown with all three candidate target peptides. **E**) Representative histograms of HA29 phage clone binding to T2 cells pulsed with 10 ug/ml B2M only (gray) or B2M + 50 ug/ml ALT (green), ILK (orange), or VLA peptides. A large shift in phage binding with ALT-pulsed cells is observed. **F**) Monoclonal phage clone HI12 shows increased binding to T2 cells pulsed with the cognate peptide (ILK) versus irrelevant peptides (VLA and ALT). **G**) Additional monoclonal phage screening via flow cytometry for VLA-specific phage yielding specific clones HV115 and HV154 are shown.

S6

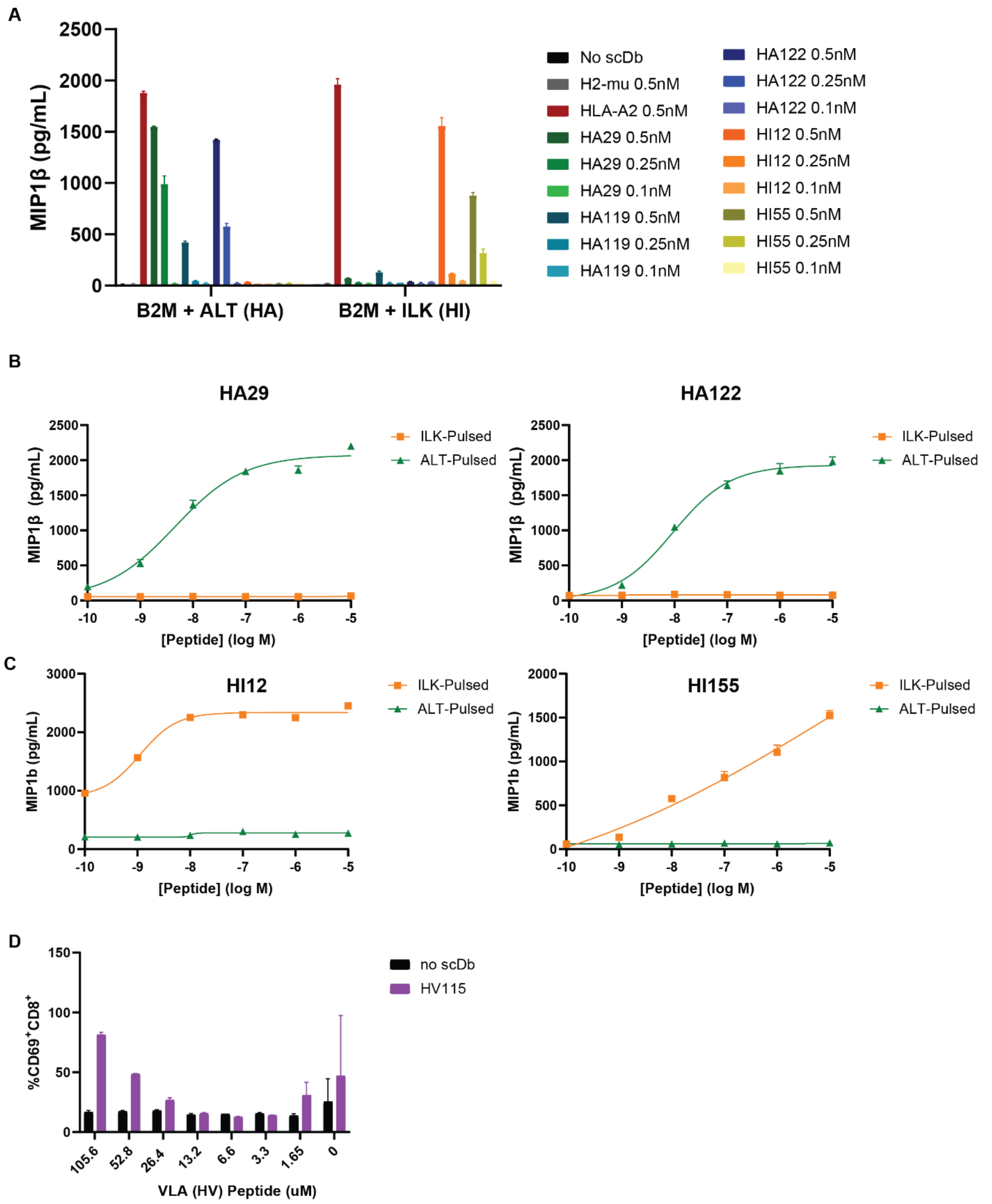

Fig. S6: Selection of HA29 and HI12-scDbs for further characterization. A) T2 cells were pulsed with 1 ug/ml ALT or ILK peptides and 10 ug/ml B2M and co-cultured with pre-activated $CD8^{+}$T cells at a 1:1 E to T ratio. HA or HI-specific scDbs were added at the indicated concentrations. After 72 hrs, supernatants from co-cultures were snap frozen and assayed for secreted MIP1β. B) T2 cells were pulsed with the ALT

or ILK peptides at the indicated concentrations and 10 ug/ml B2M. Pulsed T2 cells were incubated with pre-activated $CD8^+$T cells (1:2 E to T) and with 0.25 nM of HA29, HA122, and HI155-scDbs and .5 nM of HI12-scDb for 72 hrs. Co-culture supernatants were harvested and assayed for MIP1β. C) T2 cells were pulsed with indicated concentrations of the VLA peptide and co-cultured with pre-activated $CD8^+$T cells in the presence of 25 ng/ml HV115. CTL activation in response to decreasing doses of peptide was measuring using surface $CD69^+$ levels.

S7

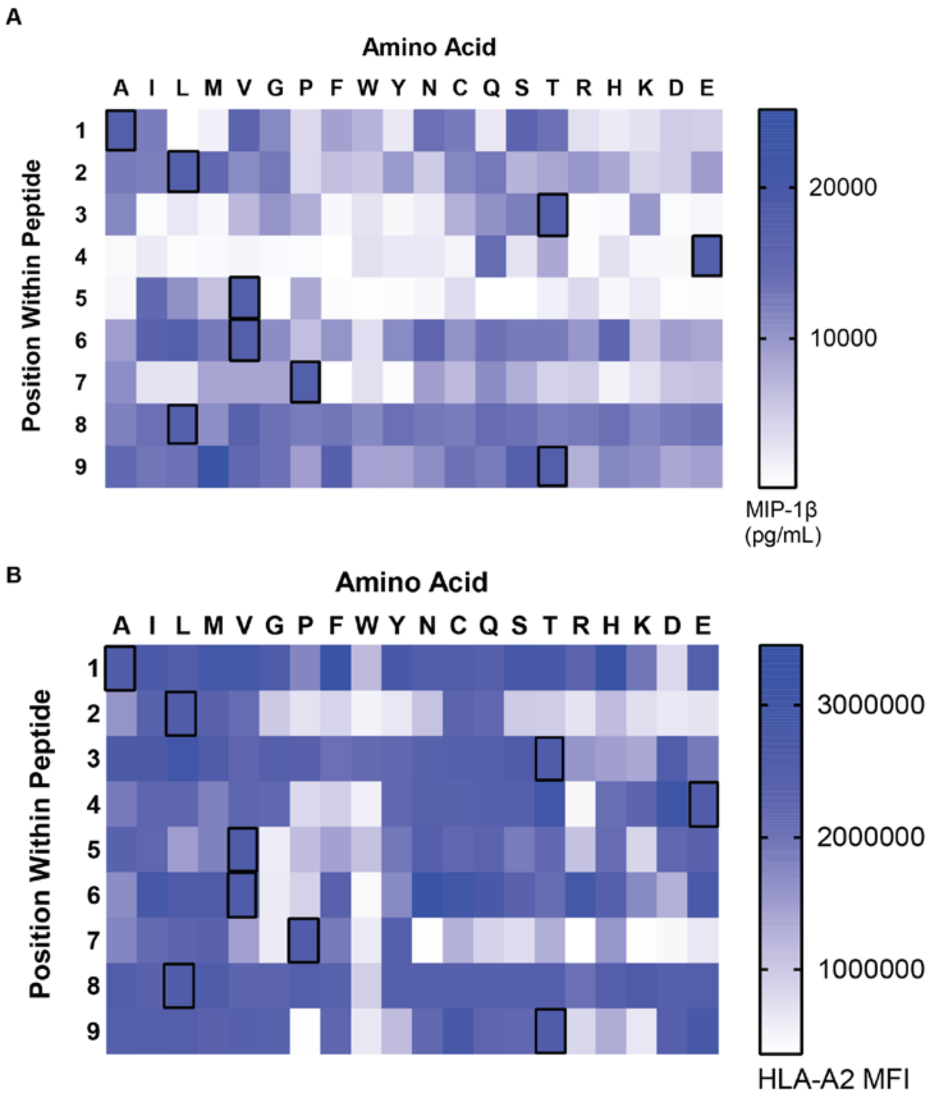

Figure S7: Characterization of HA29-scDb binding. We generated a library of 171 positional scanning variant peptides by substituting each residue of the original peptide with the 19 other possible amino acids as previously described (1, 2). T2 cells were then pulsed with each variant at 10 uM and co-cultured with pre-activated $CD8^{+}$T cells from healthy donors. A) Supernatants were assayed for MIP1β at 24 hours, and the mean of three technical replicates is plotted as a heatmap. Black boxes indicate the amino acids in the parental peptides. B) To assess for differential peptide affinity for A2 and peptide-induced A2 upregulation on T2 cells post-pulsing, we also stained for surface HLA-A2 levels.

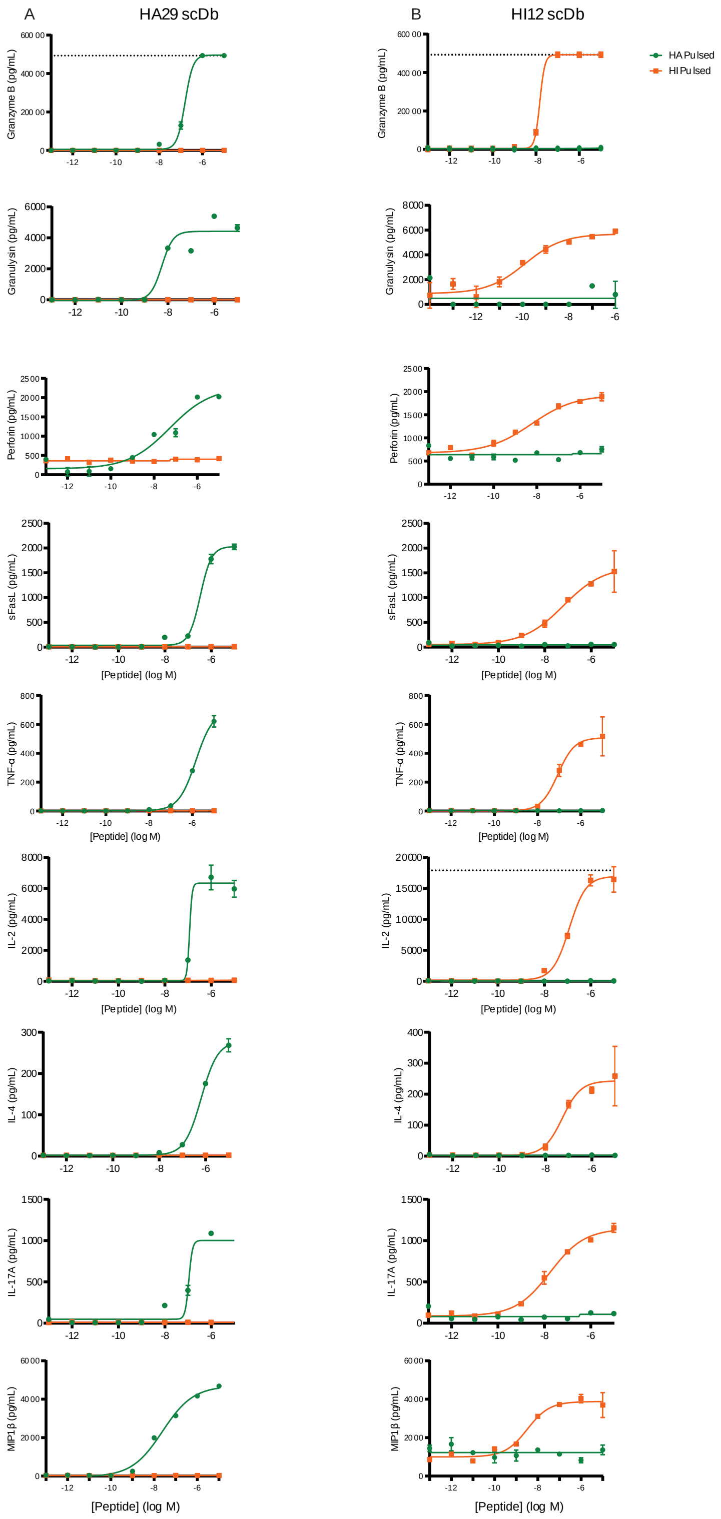

Fig. S8: HA29 and HI12-scDbs induce polyfunctional CD8$^+$T cell responses. T2 cells were pulsed with the ALT or ILK peptides at the indicated concentrations. 10 X 10^4 peptide-pulsed cells were co-cultured with 5 X 10^4 pre-activated CD8$^+$T cells (1:2 E to T) in the presence of .25 nM HA29 or HI12 scDbs for 72 hrs. Supernatants from co-cultures were assayed for the indicated effector molecules using

Legendplex. Specific activation of $CD8^+$T cells by HA29 and HI12 was observed in response to cells pulsed with the cognate but not irrelevant pMHC at doses down to the nanomolar range.

S9

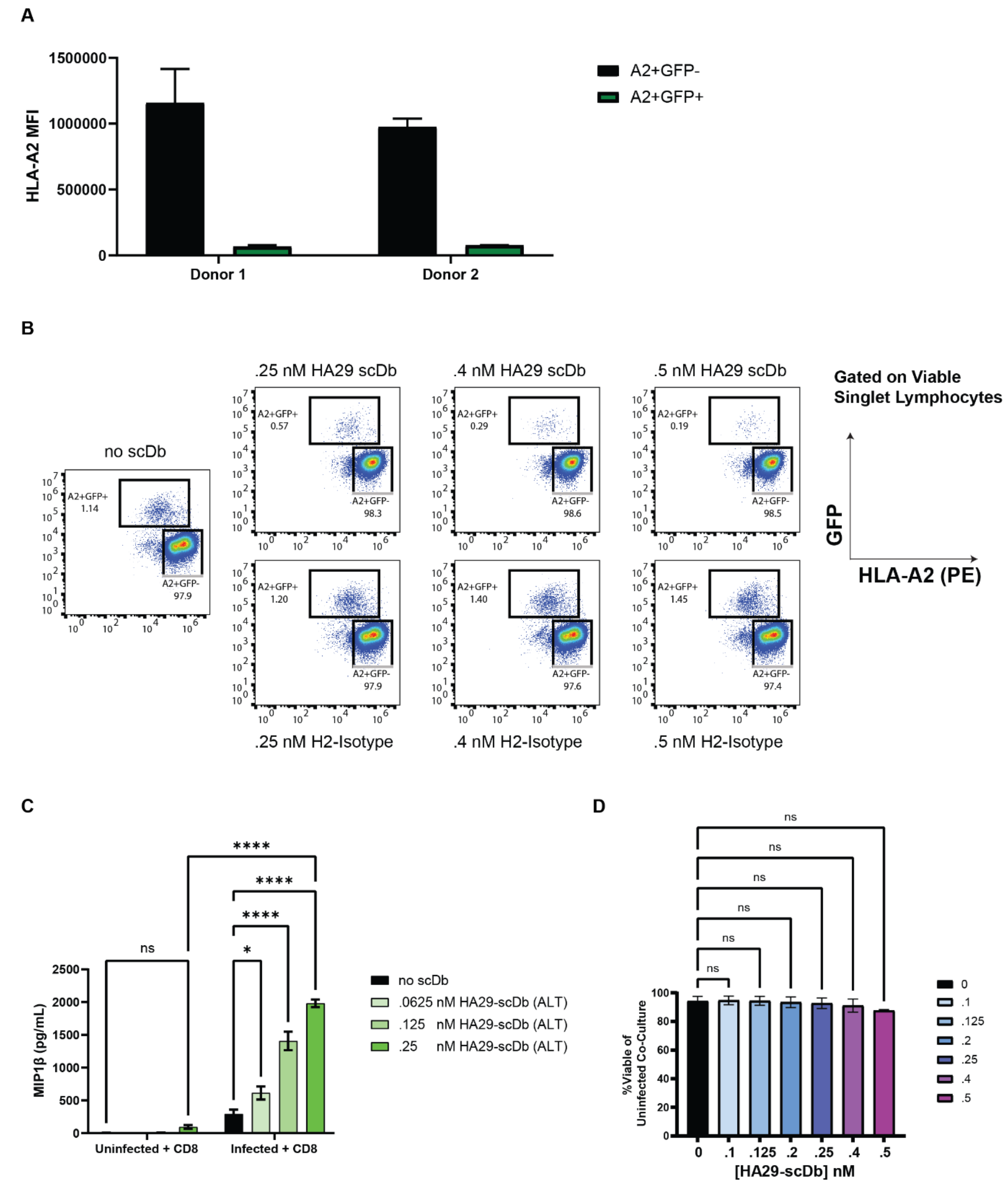

Fig. S9: HA29-scDbs induce viral suppression even in infected cells with A2 downregulation. A) Suppression assays were set up using target cells (activated CD4⁺T cells from an A2-expressing healthy donor) infected with ΔEnv NL4.3 EGFP and co-cultured with autologous pre-stimulated CD8⁺T cells for 72 hrs (see Methods) in the presence of HA29 or H2-scDb. A) MFI of surface HLA-A2 (BB7.2 clone) levels are shown from GFP+ or GFP- cells from two biological replicates of infected cells used in suppression

assays. **B**) Representative flow cytometry plots of residual viable A2+ GFP^{+} cells after 3 days of co-culture in a suppression assay, showing a dose-dependent decrease with the HA29-scDb but no decrease with the H2-scDb. **C**) Healthy donor HLA-A*02:01^{+} $CD4^{+}$T cells were activated with CD3/CD28 Dynabeads for 72 hrs and infected with ΔEnv NL4.3 EGFP. Two days post infection, infected or uninfected $CD4^{+}$T cells were co-cultured at a 1:3 E:T ratio with autologous human $CD8^{+}$T cells (15 X10^4 targets and 5 X10^4 $CD8^{+}$T cells) with the HA29-scDb at 25 ng/ml for 72 hr. scDb-induced $CD8^{+}$T cell activation was measured by MIP1β ELISAs from co-culture supernatants. **D**) %Viable cells as determined by flow cytometry in uninfected co-cultures to assess for background killing by increasing doses of HA29-scDb. Data in C show mean ± SD analyzed by two-way ANOVA followed by Tukey's Multiple Comparison Test. Data in D represent the mean ± SD of three independent experiments analyzed by one-way ANOVA followed by Dunnett's multiple comparison test. *p<0.05, **p<0.01, ***p<0.001, ****p<0.0001.

Supplementary Tables

Table S1: Predicted HLA-A*02:01 binding peptides from the HIV-1 proteome (excluding Env) for analysis by LC-DIAMS

Sequence*	Protein	Amino Acid Position†
ILGQLQPSL	Gag	60 → 68
SLQTGSEEL	Gag	67 → 75
RSLYNTIAVL	Gag	76 → 85
SLYNTIAVL	Gag	77 → 85
YNTIAVLYCV	Gag	79 → 88
NTIAVLYCV	Gag	80 → 88
VLYCVHQRI	Gag	84 → 92
SQVSQNYPI	Gag	126 → 134
TLNAWVKVV	Gag	151 → 159
QDLNTMLNTV	Gag	182 → 191
AAEWDRLHPV	Gag	209 → 218
MTHNPPIPV	Gag	250 → 258
ILGLNKIVRM	Gag	267 → 276
RMYSPTSIL	Gag	275 → 283
ATLEEMMTA	Gag	341 → 349
EMMTACQGV	Gag	345 → 353
RVLAEAMSQV	Gag	361 → 370
VLAEAMSQV	Gag	362 → 370
VLAEAMSQVT	Gag	362 → 371
AMSQVTNPA	Gag	366 → 374
FLQSRPEPT	Gag	448 → 456
FLQSRPEPTA	Gag	448 → 457
DILDLWIYHT	Nef	108 → 117
ILDLWIYHT	Nef	109 → 117
LTFGWCYKL	Nef	137 → 145
LTFGWCYKLV	Nef	137 → 146
FGWCYKLVPV	Nef	139 → 148
KLVPVEPDKV	Nef	144 → 153
GMDDPEREV	Nef	172 → 180
KMIGGIGGFI	Pol	101 → 110
RQYDQILIEI	Pol	113 → 122
ILIEICGHKA	Pol	118 → 127
TVLVGPTPV	Pol	130 → 138

NLLTQIGCTL	Pol	144 → 153
TLNFPISPI	Pol	152 → 160
KALVEICTEM	Pol	187 → 196
ALVEICTEM	Pol	188 → 196
VQLGIPHPA	Pol	245 → 253
YTAFTIPSI	Pol	282 → 290
AIFQCSMTKI	Pol	313 → 322
IYQYMDDLYV	Pol	335 → 344
YMDDLYVGS	Pol	338 → 346
HLLRWGFTT	Pol	363 → 371
FLWMGYELHP	Pol	382 → 391
YELHPDKWTV	Pol	387 → 396
VLPEKDSWTV	Pol	400 → 409
KLVGKLNWA	Pol	414 → 422
KLLRGTKAL	Pol	436 → 444
KALTEVVPL	Pol	442 → 450
KALTEVVPLT	Pol	442 → 451
ALTEVVPLT	Pol	443 → 451
ILKEPVHGV	Pol	464 → 472
ATWIPEWEFV	Pol	563 → 572
YQLEKEPII	Pol	582 → 590
PIIGAETFYV	Pol	588 → 597
IIGAETFYV	Pol	589 → 597
IIGAETFYVD	Pol	589 → 598
LALQDSGLEV	Pol	639 → 648
ALQDSGLEV	Pol	640 → 648
IVTDSQYAL	Pol	650 → 658
VLFLDGIDKA	Pol	714 → 723
RAMASDFNL	Pol	735 → 743
MASDFNLPPV	Pol	737 → 746
ASDFNLPPV	Pol	738 → 746
GQVDCSPGI	Pol	767 → 775
HLEGKVILV	Pol	782 → 790
HLEGKVILV	Pol	782 → 790
KVILVAVHV	Pol	786 → 794
LLDTGADDTV	Pol	79 → 88
YIEAEVIPA	Pol	798 → 806
LLKLAGRWPV	Pol	816 → 825
HLKTAVQMAV	Pol	886 → 895

KLLWKGEGA	Pol	955 → 963
KLLWKGEGAV	Pol	955 → 964
LLWKGEGAV	Pol	956 → 964
LLWKGEGAVV	Pol	956 → 965
KQMAGDDCV	Pol	988 → 996
TYLGRSAEPV	Rev	62 → 71
YLGRSAEPV	Rev	63 → 71
QILVESPTV	Rev	101 → 109
ILVESPTVL	Rev	102 → 110
WQVMIVWQV	Vif	5 → 13
KRLVKHHMYI	Vif	22 → 31
KISSEVHIPL	Vif	50 → 59
LVITTYWGL	Vif	64 → 72
SLQYLALAA	Vif	144 → 152
SLQYLALAAL	Vif	144 → 153
LQYLALAAL	Vif	145 → 153
KQIKPPLPSV	Vif	157 → 166
WTLELLEEL	Vpr	18 → 26
LLEELKSEAV	Vpr	22 → 31
IRILQQLLFI	Vpr	61 → 70
RILQQLLFI	Vpr	62 → 70
QLLFIHFRI	Vpr	66 → 74
PIIVAIVALV	Vpu	3 → 13
IIVAIVALV	Vpu	5 → 13
IIVAIVALVV	Vpu	5 → 14
AIVALVVAI	Vpu	8 → 16
ALVVAIIIAI	Vpu	11 → 20
LVVAIIIAI	Vpu	12 → 20
LVVAIIIAIV	Vpu	12 → 21
VVAIIIAIV	Vpu	13 → 21
IIIAIVVWSI	Vpu	16 → 25
IIAIVVWSI	Vpu	17 → 25
IIAIVVWSIV	Vpu	17 → 26
RLIDRLIERA	Vpu	41 → 50
ALVEMGVEM	Vpu	63 → 71

*No shading indicates peptides with fragmentation patterns detected by Poisson LC-DIAMS (64 peptides). Peach shading indicates peptides for which fragmentation patterns could not be identified by LC-DIAMS (43 peptides). Green shading indicates peptides that yielded fragmentation patterns by LC-DIAMS and were detected on infected (GFP+) cells (3 peptides).

†Numbers represent amino acid position relative to protein start in HXB2.

Table S2: Characteristics of each pMHC-I target

scDb Identifier	HLA Haplotype	Epitope Specificity	Epitope Location
HA	A*02:01	ALTEVVPLT	Pol (RT)
HI	A*02:01	ILKEPVHGV	Pol (RT)
HV	A*02:01	VLAEAMSQV	Gag (p24-p2)

Table S3: Sequences of V_H and V_L for relevant scDbs

scFv	VH	VL
HV115-scDb	EVQLVESGGGLVQPGGSLRLSCAAS<br>GFNVTSVQMHWVRQAPGKGLEWVA<br>MFYPDSDYTMYADSVKGRFTISADTS<br>KNTAYLQMNSLRAEDTAVYYCSRMSY<br>SSAFDYWGQGTLVTVSS	DIQMTQSPSSLSASVGDRVTITCRASQD<br>VNTAVAWYQQKPGKAPKLLIYSASFLYS<br>GVPSRFSGSRSGTDFTLTISSLQPEDFAT<br>YYCQQYYYWYPITFGQGTKVEIKRT
HI12-scDb	DIQMTQSPSSLSASVGDRVTITCRASQ<br>DVNTAVAWYQQKPGKAPKLLIYSASF<br>LYSGVPSRFSGSRSGTDFTLTISSLQP<br>EDFATYYCQQWDYHYSPVTFGQGTK<br>VEIK	EVQLVESGGGLVQPGGSLRLSCAASGF<br>NISGGSMHWVRQAPGKGLEWVAYVYPQ<br>SGNTYYADSVKGRFTISADTSKNTAYLQ<br>MNSLRAEDTAVYYCSRYYIYGLDVWGQ<br>GTLVTVSS
HA29-scDb	DIQMTQSPSSLSASVGDRVTITCRASQ<br>DVNTAVAWYQQKPGKAPKLLIYSASF<br>LYSGVPSRFSGSRSGTDFTLTISSLQP<br>EDFATYYCQQYYSSPVTFGQGTKVEI<br>K	EVQLVESGGGLVQPGGSLRLSCAASGF<br>NFSWSSIHWVRQAPGKGLEWVAQLSYY<br>SDYTNYADSVKGRFTISADTSKNTAYLQ<br>MNSLRAEDTAVYYCSRGPYYMDYWGQ<br>GTLVTVSS
H2-scDb (2)	DIQMTQSPSSLSASVGDRVTITCRASQ<br>DVNTAVAWYQQKPGKAPKLLIYSAY<br>FLYSGVPSRFSGSRSGTDFTLTISSLQ<br>P<br>EDFATYYCQQYSRYSPVTFGQGTKVE<br>IK	EVQLVESGGGLVQPGGSLRLSCAASG<br>FNVYASGMHWVRQAPGKGLEWVAK<br>IYPDSDYTYYADSVKGRFTISADTSKN<br>TAYLQMNSLRAEDTAVYYCSRDSSFY<br>YVYAMDYWGQGTLVTVSS

SI References

1. J. Douglass, *et al.*, Bispecific antibodies targeting mutant RAS neoantigens. *Sci. Immunol.* **6**, eabd5515 (2021).

2. E. H. C. Hsiue, *et al.*, Targeting a neoantigen derived from a common TP53 mutation. *Science* **371** (2021).
